# The definition and measurement of heterogeneity

**DOI:** 10.1038/s41398-020-00986-0

**Published:** 2020-08-24

**Authors:** Abraham Nunes, Thomas Trappenberg, Martin Alda

**Affiliations:** 1 Department of Psychiatry, Dalhousie University, Halifax, Nova Scotia Canada; 2 Faculty of Computer Science, Dalhousie University, Halifax, Nova Scotia Canada

**Keywords:** Neuroscience, Biomarkers

## Abstract

Heterogeneity is an important concept in psychiatric research and science more broadly. It negatively impacts effect size estimates under case–control paradigms, and it exposes important flaws in our existing categorical nosology. Yet, our field has no precise definition of heterogeneity proper. We tend to quantify heterogeneity by measuring associated correlates such as entropy or variance: practices which are akin to accepting the radius of a sphere as a measure of its volume. Under a definition of heterogeneity as the degree to which a system deviates from perfect conformity, this paper argues that its proper measure roughly corresponds to the size of a system’s event/sample space, and has units known as numbers equivalent. We arrive at this conclusion through focused review of more than 100 years of (re)discoveries of indices by ecologists, economists, statistical physicists, and others. In parallel, we review psychiatric approaches for quantifying heterogeneity, including but not limited to studies of symptom heterogeneity, microbiome biodiversity, cluster-counting, and time-series analyses. We argue that using numbers equivalent heterogeneity measures could improve the interpretability and synthesis of psychiatric research on heterogeneity. However, significant limitations must be overcome for these measures—largely developed for economic and ecological research—to be useful in modern translational psychiatric science.

## Introduction

Psychiatric discussions of heterogeneity are largely motivated by limitations of the case–control paradigm: ignorance of (A) inter-individual differences within groups, and (B) the fact that some group differences may be larger than others. These assumptions may compromise effect size estimation^1^, thereby impeding progress in understanding psychopathology and its treatment. For example, a recent study showed that clinical features could predict lithium response in bipolar disorder with an area under the receiver operating characteristic curve of 0.80 (95% CI 0.78–0.82) in a pooled international sample of 1266 subjects^2^. However, this result was limited by the fact that predictively relevant features differed based on a subject’s site of origin, which limits our ability to develop broadly generalizable treatment prediction models.

More broadly, the psychiatric literature has discussed heterogeneity in terms of meta-analysis, the combinatorial enumerations of symptom profiles (i.e., the “number of ways” disorder *X* can present)^3–7^, cluster analyses^8,9^, dimensional models^10^, concentration or inequality measures^11,12^, time-series complexity^13^, and recently in terms of “normative models”^14,15^. Unfortunately, we have neither a unified operational definition nor clear measure for this concept^16^. If we are to seriously tackle the problem of heterogeneity in psychiatry, we believe it is necessary to have a consistent, easily interpretable, and problem-agnostic framework for its definition and measurement. For example, in the case of multi-site machine learning studies, establishment of such a measurement framework could facilitate decomposition of heterogeneity into that originating from pathology-intrinsic factors and those due to between-site pooling and other sources of nuisance variation.

In this paper, we define heterogeneity as the degree to which a system diverges from a state of perfect conformity (Eliazar^17^) and undertake a focused review of more than 100 years of research concerning its measurement. Measures developed in ecology, economics, statistical physics, and more are reviewed along with some of their known psychiatric research applications. We broadly, though somewhat artificially, split these measures into those that operate on categorical or non-categorical data. We highlight that generalizable and well-behaved heterogeneity measures share a set of units known in ecology and economics as the numbers equivalent^18–22^, which allow these measures to roughly capture the “size” of a system’s sample/state space (or the number of states that a random variable can take). However, we identify several problems to be overcome before these measures can be widely applicable in modern translational psychiatric science.

## Methods

The Scopus database (which also has 100% MEDLINE coverage) was searched from inception until 16 July 2019 using the search queries detailed in the Supplementary Materials. As mentioned above, our paper is a focused review, since comprehensive exposition of heterogeneity statistics and their applications is not possible within the allotted constraints. We focus on the relevant axioms of heterogeneity measurement encountered across the literature. For each axiom, we highlight indices for which it is satisfied, then motivate additional heterogeneity axioms based on the limitations of those indices. Methodological papers were reviewed in detail if they included derivation or technical analysis of heterogeneity indices. Applied papers were reviewed if they described application of a heterogeneity index for the purpose of quantifying heterogeneity in a psychiatric research study. Reference lists of all reviewed papers, along with the bibliographies of the most prominent authors were further reviewed for additional papers. Owing to the large quantity of research discussing heterogeneity over many decades, we regrettably could not include every study encountered in our search.

## A definition of heterogeneity and measurement in categorical systems

**Fig. 1:**
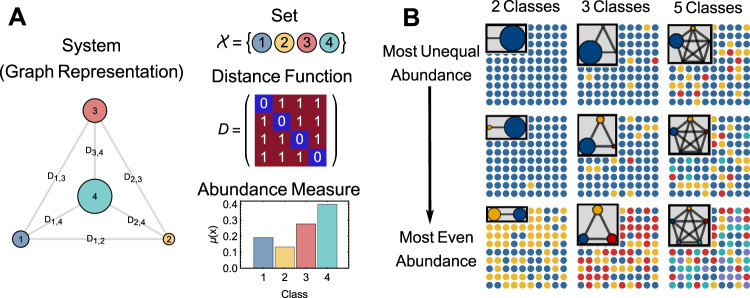
Illustration of system components and influence on heterogeneity of samples. Panel **a** depicts a categorical system comprised of a set four categories (equivalently “elements” or “partitions”) connected by undirected edges whose lengths are proportional to the distance between categories. In this case, the distances between categories are all equal (symmetric), and the within-category distance is 0 (as evident in the depicted distance matrix). These properties define the set as categorical. The size of the nodes represents their relative abundance, which is also shown in the corresponding bar chart. Panel **b** demonstrates samples from nine categorical systems with varying number of categories (2, 3, and 4) and varying levels of inequality in the abundance distribution. Systems in the upper row have the highest level of inequality in abundance, whereas the systems shown in the bottom row have perfectly even abundance distributions. Together, these plots demonstrate that heterogeneity increases with both (A) increases in the number of categories and (B) more evenly distributed abundance across categories.

A system’s heterogeneity is the degree to which it diverges from a state of perfect conformity. A “system” has three components (Fig. 1a): (A) a set, “event space”, or “sample space” ${\cal{X}}$ of distinct potential observations which one can also think of as “elements”, “partitions”, “groups”, or “categories”, (B) a measure of distance $d(x_i,x_j)$ between any two potential elements *x*_*i*_ and *x*_*j*_ in ${\cal{X}}$, and (C) a measure of abundance of each element in ${\cal{X}}$. If the abundance function sums to 1 over the entire set ${\cal{X}}$, then the abundance measure is a probability distribution.

In this section, we consider only categorical systems since they are an excellent starting point for developing intuition about the measurement of heterogeneity. Categorical systems are effectively defined by the following distance function (the discrete metric):

1 $$D_{ij} = d\left( {x_i,x_j} \right) = \left\{ {\begin{array}{*{20}{l}} 0 \hfill & {i \;= \;j} \hfill \\ 1 \hfill & {i \;\ne \;j} \hfill \end{array}} \right.$$

Like the case–control assumptions, this function states that (A) there are no inter-individual differences within a category, and (B) all categories are maximally different, thus meaning no two categories are more similar than any other two.

### Measuring heterogeneity by partition counting

A system in a state of perfect conformity is one whose event space ${\cal{X}}$ effectively has only one element. All observations from this system will be identical. All else being equal, systems that deviate further from perfect conformity will thus have larger event spaces (Fig. 1b). Partition counting methods work on the assumption that the size or “cardinality” of ${\cal{X}}$—the number of distinct partitions or elements it contains—measures that system’s heterogeneity.

Partition counting methods are often used to quantify a disorder’s clinical heterogeneity by the number of criteria-satisfying symptom combinations^3–7,23,24^. Here, one assumes that the “system” is the disorder in question. For each diagnosis, the set ${\cal{X}} = \left\{ {1,2, \ldots ,n_c^ \ast } \right\}$ consists of a total of $n_c^ \ast$ (the asterisk denotes that this is the “true” value, which may or may not be known) categorically unique symptom combinations or “presentations”. Estimating $n_c^ \ast$ amounts to estimating the system’s heterogeneity. The next few sections will describe several approaches for this estimation problem.

#### Combinatorially estimating an upper bound for $n_{\mathbf{c}}^ \ast$

Many studies estimate an upper bound for $n_c^ \ast$ using combinatorial methods. In these cases, one is not obtaining $n_c^ \ast$ from empirical data; rather, one directly calculates the total number of unique configurations that may be realized by that categorical system. Hence, this is an upper bound on $n_c^ \ast$ since empirical data could not exceed the computed value. For example, a diagnosis of generalized anxiety disorder (GAD) under the Diagnostic and Statistical Manual of Mental Disorders (5th edn)^25^, requires three or more of six symptoms. If we denote the total number of available symptoms as *N* and the number of required symptoms as *K*, the number of unique symptom combinations is

2 $$S\left( {N,K} \right) = \mathop {\sum}\limits_{k = K}^N {\frac{{N!}}{{k!\left( {N - k} \right)!}}}$$

One calculates that GAD has at most $S\left( {6,3} \right) = 42$ unique presentations. Similarly, one can verify that for borderline personality disorder $S(9,5) = 256$, for catatonia $S(12,3) = 4017$. For major depressive disorder (MDD), which has mandatory symptoms of either low mood or loss of interest, one can show that there are 227 symptom combinations.

#### Estimating *n*_*c*_ empirically from data

Zimmerman et al.^4^ found a total of 170 unique symptom combinations in a survey of 1500 MDD patients, suggesting that 25% of theoretical symptom combinations do not occur. Similarly, Park et al.^5^ found 119 unique combinations in 853 subjects further highlighting that empirical estimates of $n_c^ \ast$ are important complements to combinatorial enumeration. Unfortunately, any sample short of a complete census will underestimate $n_c^ \ast$, particularly if many of the categories in ${\cal{X}}$ are rare.

The simplest, but most biased (lower limit), estimator of $n_c^ \ast$ is the observed richness (also known as species richness to ecologists)^16,26^, which is the observed number of categories in the sample. We denote this quantity as ${\Pi}_0 = n_c$ (the lack of asterisk denotes it is an estimate).

A less biased approach for estimating $n_c^ \ast$ is to compute a lower bound^26,27^, using the Chao estimators. These indices, which are standard in ecology, use information about the frequency of rare categories to speculate on how many further rare categories may exist who have not yet been sampled. If we denote *f*_*K*_ as the number of categories observed only *K* times, then the corresponding Chao estimator is as follows^28^:

3 $${\mathrm{Chao}}_1\left( f \right) = \left\{ {\begin{array}{*{20}{l}} {{\Pi}_0 + \frac{{f_1^2}}{{2f_2}}} \hfill & {f_2\, > \,0} \hfill \\ {{\Pi}_0 + \frac{1}{2}\left( {f_1\left( {f_1 - 1} \right)} \right)} \hfill & {f_2 \,= \,0} \hfill \end{array}} \right..$$

**Fig. 2:**
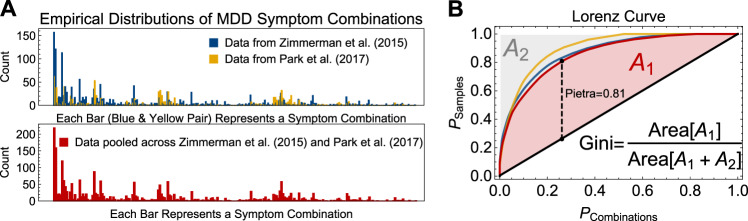
Illustration of the distribution of major depressive disorder symptom combinations and analysis of inequality via Lorenz curves. **a** Distribution of symptom presentations in patients with major depressive disorder as reported by Zimmerman et al.^4^ and Park et al.^5^ (data extracted from their published tables). **b** Lorenz curves for the empirical distributions shown in (**a**). Curve colors are matched between panels. In this case, the Lorenz curve demonstrates the proportion of symptom combinations $\left( {P_{{\mathrm{Combinations}}}} \right)$ that account for at least $P_{{\mathrm{Samples}}}$ proportion of observed presentations in the datasets. The diagonal (black) line represents the line of perfect equality, which would occur only if all symptom combinations accounted for the same proportion of observed presentations. The closer a Lorenz curve is to the upper corner, the more inequality exists in the abundance distribution, which in this case would indicate greater homogeneity of symptom presentations. Geometric calculation of the Gini coefficient and Pietra indices is also demonstrated. The Gini index is the ratio of (A) the area between the Lorenz curve and the line of perfect equality to (B) the total area above the Lorenz curve. The Pietra index is the maximum distance from the Lorenz curve to the line of perfect equality, and represents the proportion of observations that would need to be transferred from the most common to the least common symptom combinations in order to reach the line of perfect equality.

The observed richness values reported by Zimmerman et al.^4^ and Park et al.^5^ underestimate the true number of MDD presentations. After abstracting the presentation frequency tables from these papers (Fig. 2a), we used the Chao estimator to recalculate lower bound estimates on the number of MDD symptom combinations. In the Zimmerman et al.^4^ data, this was 189.8 (95% confidence interval, CI [189.3, 190.2]), compared to 144.1 (143.4, 144.9) for the Park et al.^5^ data, and 200.6 (200.4, 200.9) in the pooled sample. Thus, the heterogeneity of symptom combinations in MDD may be larger than previously estimated using empirical data.

Observed richness and the Chao estimator have been used to quantify gut microbiomic heterogeneity in psychiatric samples, finding no difference between healthy controls and males with attention deficit-hyperactivity disorder (ADHD)^29^, but lower microbiome diversity in patients with MDD^30^.

The Chao estimators are notably related to capture–recapture methods^26,31^, which estimate the size of difficult-to-sample population by examining overlap in repeated samples. Applications include estimation of the prevalence of alcohol-related disorders^32^, opioid addiction^33^, and other conditions^34–40^. Krebs^41^ reviews these approaches.

#### Limitations of partition counting approaches

Partition counting methods ignore abundance inequalities. For example, imagine 99.999% of all patients showed a single presentation of MDD, with the remaining 0.001% spread across the other 226 symptom combinations. This system is effectively close to perfect conformity, yet partition counting methods would nonetheless overestimate a heterogeneity value of 227 presentations.

### Measures accounting for inequality in category abundance

Consider a scenario in which 99.999% of all patients have the same presentation of MDD, with the remaining 0.001% evenly spread across the other 226 symptom combinations. In this section, we compute how far this system diverges from perfect conformity given the highly skewed abundance distribution. We restrict our search to those indices that satisfy the axiom of monotonicity to set size (heterogeneity must increase if a system’s event space grows in size), but also further satisfy the axiom of transfers^42,43^. That is, any transfer of abundance from a more abundant category to any less abundant category (thereby making the abundance distribution more even) must increase heterogeneity. This is sensible, since in the opposite scenario—progressively stacking all abundance onto a single category—would push the system toward perfect conformity.

The most common of these heterogeneity indices are the Tsallis family entropies^44^, most notably the Shannon entropy^45^,

4 $$H({\mathbf{p}}) = - \mathop {\sum}\limits_{i = 1}^{n_c} {p_i{\mathrm{log}}\;p_i}$$

which measures the average amount of uncertainty in the system. If the logarithm is taken with base 2, then Shannon entropy gives the average number of yes/no questions required to classify an observation from the system.

The Gini–Simpson index (GSI)^46^ is another historically important entropy:

5 $${\mathrm{GSI}}\left( {\mathbf{p}} \right) = 1 - \mathop {\sum }\limits_{i = 1}^{n_c} p_i^2$$

The GSI is the probability that two observations from our system (sampled with replacement) will belong to different categories.

The GSI is related to a concentration index commonly attributed to Simpson^47^ or Herfindahl^48^:

6 $${\mathrm{Simpson}}\left( {\mathbf{p}} \right) = \mathop {\sum }\limits_{i = 1}^{n_c} p_i^2 = 1 - {\mathrm{GSI}}\left( {\mathbf{p}} \right)$$

which gives the probability that two samples from our system will belong to the same category. Psychiatric researchers have used this to measure the homogeneity of physicians’ and health systems’ prescription repertoires^11,12^.

Olbert et al.^3^ used the GSI and a normalized version of the Shannon entropy to empirically quantify symptom heterogeneity in MDD and PTSD. Using data from $n_s = 84,103$ subjects with MDD in the National Comorbidity Survey Replication (NCS-R)^49^, they found an observed richness of 137 unique symptom combinations. The probability of sampling two individuals with MDD whose symptom profiles were different (i.e., the GSI) was 0.96, suggesting a high degree of symptomatic diversity in MDD. However, their Shannon entropy index (with base 2) was 3.9 bits, meaning that approximately four yes/no questions could precisely identify a typical subject’s specific symptom profile given only knowledge of their MDD diagnosis.

If one accepts that the GSI and Shannon entropy are both measures of heterogeneity, then the results obtained by Olbert et al.^3^ are puzzling. On the one hand, the GSI suggests that most pairs of MDD patients will have different symptom profiles (GSI = 96%). Conversely, the Shannon entropy amounted to 55% of its theoretical maximum (3.9 of 7.09 bits), suggesting less heterogeneity than the GSI, illustrating the problem of multiple meanings between entropic-based heterogeneity indices. Synthesizing the results from such indices with different meanings can be challenging, and thus we seek measures with conceptually standard units.

Entropy-based heterogeneity indices also fail to satisfy the axiom of replication (also known as the replication principle in ecology)^18,21,22,50^. The replication principle states that if we pool *K* completely unique independent systems with equal amount of heterogeneity, *h*, then the heterogeneity should measure *K* × *h*. Jost^22^ noted this is akin to merging two spheres, each with volume *V*; the resulting volume of the pooled sphere should be 2*V*, which would not be the result if we treated the sphere’s radius (a mere index of volume) as a measure.

### Numbers equivalent measures of heterogeneity

One family of indices satisfy the replication principle, and its units are the same units as partition counting methods: the (effective) number of distinct elements in an event space. We call this family the Rényi heterogeneity since it is the exponential function of Rényi entropy^51^,

7 $${\Pi}_q\left( {\mathbf{p}} \right) = \left( {\mathop {\sum }\limits_{i = 1}^{n_c} p_i^q} \right)^{\frac{1}{{1 - q}}},$$

also known as the Hill numbers in ecology^20^, and the Hannah–Kay indices in economics^52^, with elasticity parameter *q* ≥ 0. When *q* = 0, the abundances are ignored, and we recover the observed richness:

8 $${\Pi}_0\left( {\mathbf{p}} \right) = \mathop {\sum }\limits_{i = 1}^{n_c} p_i^0 = n_c$$

Taking the limit as *q* → 1 yields the exponential of the Shannon entropy, which is the perplexity^53^ or the effective number of typical categories in the system:

9 $${\Pi}_1\left( {\mathbf{p}} \right) = e^{ - \mathop {\sum }\nolimits_{i = 1}^{n_c} p_i{\mathrm{log}}\;p_i}.$$

At *q* = 2, we have the inverse Simpson concentration^16^,

10 $${\Pi}_2\left( {\mathbf{p}} \right) = \frac{1}{{\mathop {\sum }\nolimits_{i = 1}^{n_c} p_i^2}}$$

which is the effective number of common categories in the system, known to political scientists as the effective number of parties^54^. This measure has been used to estimate the effective number of common bacterial species in the microbiome of patients with MDD^30^.

The units of Rényi heterogeneity are known as numbers equivalent^18,19,55^. These units can be intuitively understood as follows: for any system *A* with a given abundance distribution, we can find a “hypothetical” categorical system *B* whose abundance distribution is perfectly even, and whose heterogeneity is equal to that of *A*. The number of partitions in this “equivalent” system *B* serves to measure the heterogeneity of *A*. Numbers equivalent allow us to account for inequality in the abundance distribution while retaining the units of set size.

Rényi heterogeneity satisfies the axiom of replication (see Supplementary Appendix A for the proof). Recall that if we pool two equally heterogeneous systems that are completely distinct (i.e., no overlap in their event spaces), we are doubling the amount of heterogeneity. Any true measure of heterogeneity should thus also double under this circumstance. Only the Rényi heterogeneity family of indices will reflect this doubling, which is the reason why ecologists refer to it as the “true diversity”^56^. Satisfaction of the replication principle alone (in addition to the axioms previously identified) suffices to justify the Rényi family as superior to other heterogeneity indices. Any consistent argument against this point would be compelled to also argue that a sphere’s radius is a measure of its volume, since they are monotonically related, but only volume itself obeys the replication principle.

The axiom of decomposability is also satisfied^56^. That is, if a system is composed of *K* pooled groups, then the overall heterogeneity (known as *γ*-heterogeneity) must be decomposable into within- and between-group components (“*α*-heterogeneity” and “*β*-heterogeneity”, respectively). Decomposition of Rényi heterogeneity satisfies some important criteria that are beyond are present scope (see Jost^56^). Heterogeneity decomposition is commonly employed in meta-analysis (via the *I*^2^ statistic), albeit not using units of numbers equivalent.

### Inequality indices for comparing heterogeneity of differently sized sets

It is sometimes useful to measure abundance inequality independently of the event space size (but see Jost^57^ for counterpoints). For instance, let each individual in a population be a “partition” in our system, and the abundance measure his or her share of the total populations’ wealth. If we collect such data from two populations of different sizes and compare their Rényi heterogeneity values, our results will be confounded by the population sizes; the larger population will tend to have a higher heterogeneity despite potentially having more wealth inequality. For this reason, isolated measures of inequality tend to be invariant to the size of the event space: a property known as non-extensivity or the population principle^58,59^. There are two main approaches to compute these inequality measures: methods based on the Lorenz curve^60^, and derivations based on normalization of the Rényi heterogeneity^57^.

The Lorenz curve^60^ represents the percentage of total abundance in a system belonging to the top *x*% of categories. For example, when examining the distribution of abundance across presentations of MDD^4,5^, the Lorenz curve (shown in Fig. 2b) shows that 50% of all observed samples were attributable to only 7.1% of MDD symptom combinations in the pooled sample. Several summary indices can be computed from the Lorenz curve, such as the Gini coefficient (which we also discussed above)^46^ or the Pietra index^61^. Several Lorenzian inequality indices are well reviewed elsewhere^59,62^.

The distribution and utilization of psychiatric resources has been quantified with Lorenz curves^63–66^, although other questions have also been addressed^67–70^. However, (direct) Lorenzian inequality analysis is univariate, which limits applicability to modern translational psychiatric research.

An alternative to the Lorenzian approach is to define a measure of “evenness” (conceptually the opposite of inequality) by expressing Rényi heterogeneity relative to its theoretical maximum (the observed richness):

11 $$\tilde {\Pi}_q\left( {\mathbf{p}} \right) = \frac{{{\Pi}_q\left( p \right)}}{{n_c}}.$$

This is based on the more general concept of a diversity profile discussed in detail elsewhere^56^. The range of Eq. (11) is the $(0,1]$ interval, and it can be used to derive many well-known inequality indices such as Heip’s index^71^, Pielou’s J^72^, and the generalized entropy index (GEI)^58,59^, which is itself generalizes several important indices^43,73,74^. This approach has not clearly been used for inequality measurement in psychiatry.

#### Limitations of categorical heterogeneity measures

The main problem with categorical heterogeneity measures are the assumptions of categorical data. First, categories to which one’s data belong must be (A) known a priori and (B) scientifically valid. In some cases, this will be more problematic than in others. For example, defining species as categories (as ecologists do) is likely of greater validity than defining the categories as DSM-5 diagnoses.

Second, one must assume that all members of the same category are identical in every way, and that all between-category differences are equal. These assumptions about the within- and between-category dissimilarity are surely violated in most psychiatric research applications. For example, the analyses of Zimmerman et al.^4^ and Park et al.^5^ (and our reanalysis thereof) did not account for the fact that different presentations will share symptoms in common. Clearly, these are not categorical data.

Despite these limitations, categorical heterogeneity measures—and particularly the Rényi heterogeneity family—have advantages related to interpretation. The “size” of a system’s event space is an intuitive and principled measure of deviation from perfect conformity. In our MDD example, we spoke in terms of the easily understandable units of “number of symptom combinations” rather than of bits or probabilities. Rényi heterogeneity also respects the replication principle and can be decomposed into within- and between-group components. We now seek a measure that retains these useful properties without restriction to categorical data.

## Non-categorical heterogeneity indices

The elements of non-categorical systems vary in the degree to which they are similar to each other. Non-categorical heterogeneity indices include those that split the observations into categories defined a priori, and those that either (A) do not assume such a stratification at all or (B) attempt to learn it from the data.

### Methods requiring a priori stratification

These methods first split observations from a system into one of *n*_*c*_ predefined categories (e.g., diagnoses or species). However, (A) the within-category distance can exceed 0 (e.g., acknowledging that “tall” people still vary in height), and (B) the distance between pairs of categories can be asymmetrical (e.g., lobsters are “further” from elephants than they are from crabs).

The experimenter must choose a relevant distance measure, which will significantly impact the heterogeneity estimates. Returning to our reanalysis of the MDD symptom combination data^4,5^, we clarify that each of the 227 unique symptom combinations is a distinct category in the event space ${\cal{X}}$. However, we now specify the dissimilarity between symptom combinations *x*_*i*_ and *x*_*j*_ using the Jaccard distance^75^:

12 $$D_{ij} = 1 - \frac{{\# {\mathrm{Symptoms}}\;{\mathrm{occurring}}\;{\mathrm{in}}\;{\mathrm{both}}\;x_i\;{\mathrm{and}}\;x_j}}{{\# {\mathrm{Symptoms}}\;{\mathrm{occurring}}\;{\mathrm{in}}\;{\mathrm{either}}\;x_i\;{\mathrm{or}}\;x_j}}$$

which takes values between 0 (complete overlap of symptoms) and 1 (no symptoms in common). This results in a 227 × 227 matrix, **D**, of distances between symptom combinations.

To quantify heterogeneity, **D** must be summarized into a single non-negative value. The most common approaches are related to Rao’s Quadratic Entropy (RQE)^76^,

13 $$Q\left( {{\mathbf{D}},{\mathbf{p}}} \right) = \mathop {\sum }\limits_{i = 1}^{n_c} \mathop {\sum }\limits_{j = 1}^{n_c} D_{ij}p_ip_j$$

which is the average pairwise distance between categories in the system. For our present example, we have an RQE = 0.35 for the Zimmerman et al.^4^ data, RQE = 0.38 for the Park et al.^5^ data, and RQE = 0.37 in the pooled sample. Note that the RQE of one of the subsets is greater than the pooled sample’s heterogeneity^5^, which is problematic, since pooling non-identical systems should monotonically increase the overall heterogeneity. By using a different distance metric (the Hamming distance), this problem disappears; we obtain RQE estimates of 2.89^4^, 3.04^5^, and 3.05 (pooled). How are we to compare these estimates which are on ostensibly different scales? Moreover, is one set of estimates “more correct” than the other?

Researchers have thus sought to develop RQE-based measures with units of numbers equivalent since they do not appeal to the units of a given distance metric^50,77–80^, and will obey the replication principle^50,79^. Unfortunately, current RQE-based numbers equivalent measures have some idiosyncratic limitations that virtually obviate their psychiatric research applicability. For instance, the functional Hill numbers^77^ become insensitive to distance between categories when they are equally abundant (Supplementary Appendix B). We are thus unaware of any studies in the psychiatric literature that employ non-categorical heterogeneity indices with a priori stratification.

The RQE-based heterogeneity indices are unfortunately dependent on the imposed stratification, which will be problematic when strata are unreliable or invalid (such as the case in which strata are DSM-5 psychiatric diagnoses).

**Fig. 3:**
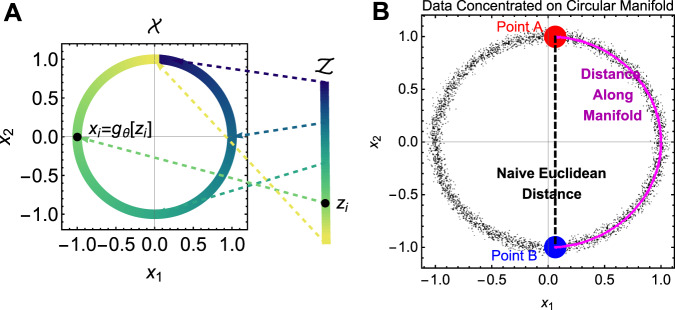
Demonstration of how data in an observable space ${\cal{X}}$ can be concentrated along a manifold (here just a curve). Panel **a** shows how the curve is simply an image of a latent space ${\cal{Z}}$ projected through a generator function $x_i = g_\theta (z_i)$. Panel **b** demonstrates noisy data along the circular curve illustrated in (**a**). Measurement of the Euclidean (straight-line) distance between points A and B implies traversal across a region of ${\cal{X}}$ in which no data lie. The correct approach is instead to measure distance with respect to the data’s manifold of support.

There is also a problem with defining the distance metric a priori. The distance metric chosen determines which paths between points A and B in the data space are “allowed”. An appropriate distance metric should allow only realistic paths between these points (Fig. 3). For example, the straight-line distance between Toronto and Tokyo is irrelevant to travelers, since that path cannot be traversed. In that vein, many real-world data are thought to be embedded on lower dimensional manifolds in the data space^81^. In such cases, the distance between points should be measured on paths along that manifold, which may be curved. Since the manifolds of support will vary between datasets, it is unlikely that predefined distance metrics (such as a global Euclidean distance) will accurately describe the dispersion of one’s data. To our knowledge, this problem remains unaddressed in the heterogeneity measurement literature.

### Methods that do not require a priori stratification

There are three main approaches to quantify heterogeneity when no compelling a priori stratification exists: (A) treating heterogeneity as the “volume” of a space that completely encloses one’s data points, (B) clustering-based methods, and (C) dendrogram-based methods.

#### Heterogeneity as a convex hull volume

**Fig. 4:**
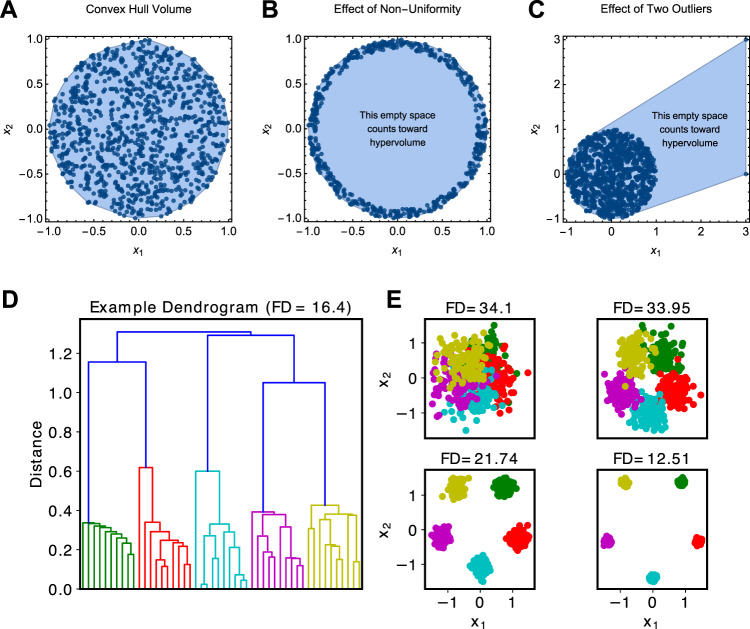
Illustration of convex hull and dendrogram-based heterogeneity indices for non-categorical systems. Panel **a** illustrates the basic concept of a convex hull on synthetic 2-dimensional data. The volume of the hull is taken as an index of heterogeneity. Panel **b** shows one problem with the convex hull method, which occurs when data lie along a lower dimensional surface (here just a curve). In this example, the data are all concentrated along the outer border of the hull, leaving the core unoccupied. However, the convex hull volume index will nonetheless count the empty space toward the heterogeneity value. Panel **c** illustrates the effect of outliers on convex hull volume. Since a convex hull is found by creating a “shell” around one’s data, outlying points will expand this shell in ways that leave much of the convex hull empty (though still counting toward the heterogeneity value). Panel **d** shows the dendrogram computed using agglomerative clustering for a simple mixture of five 2-dimensional (2D) Gaussians. The functional diversity (FD) measure, shown in the title, is the sum of all branch lengths in this tree. Panel **e** shows a simple simulation with five 2D Gaussians (standardized to lie within the bounds [−1.5, 1.5] in both axes) that were progressively separated further. One can appreciate that the FD measure decreases as the distributions become more distinct. This is the opposite effect demonstrated by the convex hull volume, insofar as FD increases as the space becomes more densely populated with data points.

Roughly speaking, the space enclosed by the smallest perimeter around all pairwise paths in one’s data is a convex hull. The volume of this space is sometimes used as a heterogeneity index^82,83^, but if data are not distributed uniformly within the convex hull, heterogeneity will be overestimated (Fig. 4a–c). We know of no psychiatric study using convex hull volume to quantify heterogeneity.

#### Methods based on clustering and dendrogram construction

Psychiatric studies often characterize a heterogeneity as the number of latent categories in some data. For example, cluster analytic studies of MDD have reported discovery of between 1 and 5 strata (depending on the data), although these groups are qualitatively inconsistent^84^. Similarly, cluster analyses in several psychopathologies^8,9,85–110^ have returned proposals for various stratifications, with heterogeneity implicitly “measured” by cluster counting.

Aside from sensitivity to the clustering method, there are three other prominent limitations of cluster counting. First, cluster counting is a variation on observed richness, since it does not capture inequality in the distribution of subjects across clusters. Second, the clusters themselves are consequently assumed to be internally homogeneous and maximally dissimilar from the other clusters. Finally, statistically optimal clustering portends neither biological or scientific validity. To address this, many reports have validated their inferred clusters using external data^111–114^. Notwithstanding, there remain several open areas for improvement in measuring heterogeneity using cluster analysis, particularly with respect to (A) evaluation of whether a clustering approach (i.e., mapping some data onto a categorical space) is appropriate for some data in the first place, and (B) accounting for uncertainty in the number of clusters, which overlaps with our above discussion of partition counting methods.

An alternative approach involves measuring heterogeneity by first performing agglomerative clustering on a pairwise distance matrix, and then computing the sum of all branch lengths in the resulting hierarchical tree (also known as a “dendrogram”; Fig. 4)^115,116^. It may be possible to compute an effective number from dendrogram-based analyses^117^. Whereas the convex hull approach defines heterogeneity by the most extreme points in a dataset, the dendrogram-based methods are sensitive to the density of sample space coverage. Unfortunately, this will create a problem if there are truly multiple groups in one’s data, since the dendrogram-based heterogeneity index increases if the groups’ feature distributions become more similar (Fig. 4e). To our knowledge, there are no applications of dendrogram-based heterogeneity measures in the psychiatric literature, although gene co-expression studies are ostensibly immediate targets for these indices^118–120^.

Normative modeling is a recent development for characterizing heterogeneity^15^. Briefly, this approach evaluates the degree and uncertainty with which individual subjects deviate from a distribution of normal variation, assuming that pathological states tend to deviate more extremely. Applications include (predominantly neuroimaging) studies of autism^121,122^, ADHD^14,123,124^, schizophrenia and psychosis^125,126^, bipolar disorder^125^, and neurocognitive disorders^127,128^. To our knowledge, no study employing this method has offered a measurement of heterogeneity. Thus, it would be of great interest to develop numbers equivalent measures applicable within the normative modeling framework.

### A note on meta-analytic heterogeneity

Standard meta-analytic methods employ parametric indices of heterogeneity on non-categorical spaces^129^. A full discussion of this (likely familiar) topic is beyond our present scope, but in Supplementary Appendix C we demonstrate that meta-analytic heterogeneity can potentially (A) be expressed in the units of numbers equivalent, and (B) decomposed into within and between-group components, such that the latter component has units of “the effective number of distinct study effects”.

### A note on heterogeneity indices for time-series and dynamical systems

We briefly discuss measurement of heterogeneity in time-series data by indices often known as “complexity” measures. Psychiatric studies have employed geometric indices (such as the Largest Lyapunov Exponent and recurrence plot analysis)^130,131^, entropic indices (such as Kolmogorov-Sinai or metric entropy^132^, approximate entropy^133^, sample and multiscale entropies^134–136^, and Lempel-Ziv complexity^137,138^), and various fractal dimension indices to electrophysiological, functional neuroimaging^139^, and other time series^134,135,140^. Numerous clinical and technical reviews of these indices exist^13,133,141–145^, so we merely note that numbers equivalent can also be of use in this domain. For example, the Shannon entropy of a time series’ normalized power spectrum, also known as spectral entropy^142^, can be easily converted to the “effective number of typical frequencies” using Eq. (9); reporting such a measure in terms of the effective number of frequency bands makes interpretation and criticism more clear. If one reports that a time series of mood recordings contains an effective number of three frequency bands, we may more readily appraise whether such information is useful, and how so. With such clear units, one may decide that indices expressing the “effective number of trajectories” or “effective number of ‘mood states’” might be more desirable.

Many conditions have been studied under this paradigm using various modalities^144,146,147^. For instance, our group has investigated the temporal dynamics of mood in patients with bipolar disorder. The overall complexity of mood fluctuations is ostensibly reduced among probands and their unaffected relatives^134,135^, with increases observed within 60 days of a mood episode^140^. Unfortunately, on the whole, it can be difficult to interpret time-series complexity between studies, since the large number of indices (each with their own units), experimental conditions, data modalities, and disorders can interact to yield various conclusions.

### Limitations of non-categorical heterogeneity indices

Non-categorical heterogeneity indices are predominantly based on RQE^76^. Unfortunately, the requirement of selecting a distance measure a priori introduces problems comparing RQE across datasets with different distance metrics. Moreover, for real-world datasets, standard methods of measuring distance will likely fail to respect data’s true underlying geometry. This problem will be shared by dendrogram-based methods and clustering-based approaches that demand pre-specification of a distance measure.

The units of RQE-based heterogeneity indices are also not clearly appropriate for thinking about heterogeneity, although one may correctly argue that heterogeneous systems have larger overall amounts of pairwise distance between its elements^148^. Plainly, these indices violate the replication principle which leads to unintuitive scaling behaviors^78,79^. Numbers equivalent transformations of RQE also have further limitations that preclude their application to psychiatric research problems. First, they continue to require prespecified categories on the data as well as prespecified distance measures. Second, they have problematic idiosyncratic limitations such as insensitivity to distance under equally abundant categories^77^.

Meta-analytic heterogeneity is at present quantified by variance, which we show in Supplementary Appendix C to violate the replication principle.

Time-series complexity measures, too, can be difficult to interpret and synthesize. In many cases, time-series complexity measures based on numbers equivalent could simplify interpretation. In the case of longitudinal self-ratings of mood, for example, reporting heterogeneity as “the effective number of mood states” could meaningfully improve the broader clinical interpretability of such results. However, no such study has heretofore reported time-series heterogeneity in numbers equivalent, and so its evaluation in that context remains an interesting future direction.

## Discussion and conclusions

This paper defined heterogeneity as the degree to which a system diverges from perfect conformity, and measures it by the effective size of a system’s event space. A large number of indices have been discovered (and rediscovered) independently, the most important of which our paper compared in a format that (A) highlighted the important axiomatic properties of heterogeneity measures, and (B) motivated additional axioms/properties based on the limitations of indices already discussed. Ultimately, measures in units of numbers equivalent were found to resolve many limitations of other indices. Although each index has valuable features, their large variety of units and differences in mathematical properties impede (A) their synthesis across studies and (B) their broader interpretability. However, we demonstrated that numbers equivalent measures of heterogeneity—known in different fields as the Rényi heterogeneity, Hill numbers, or Hannah–Kay indices—are cross-cutting measures that can potentially express the heterogeneity of any system as the size of an equally heterogeneous uniform event space. These measures satisfy most heterogeneity axioms (especially the replication principle, which ensures that the Rényi heterogeneity scales linearly with changes in the true underlying heterogeneity) and are standard measures of ecological biodiversity yet remain relatively absent from the psychiatric literature. That being said, we also showed that several limitations remain, particularly for measurement of heterogeneity in non-categorical systems. In this section, we re-highlight some of the roadblocks to their psychiatric implementation and future directions of research. Establishing a consistent, interpretable, and well-behaved approach for measuring the amount of heterogeneity in a system will be necessary to facilitate rigorous quantitative research on the causes and impact of heterogeneity in psychiatric research.

There are several conceptual obstacles remaining for implementation of numbers equivalent-based heterogeneity measures in the psychiatric literature. Heterogeneity is often discussed in the psychiatric literature, but it is rarely discussed as a concept independent of its causes and consequences. It is also common for studies to note that heterogeneity in clinical conditions can be broken down along multiple dimensions, and proposing methods for doing so^123,125,149^. However, this is not the same as measuring the absolute quantity of heterogeneity, which requires precise definition of units and establishment of some level of calibration (as we demonstrated for the Rényi family through axiomatic review). Heterogeneous systems have many correlated properties that, in the absence of precise definition, could easily be mistaken for heterogeneity itself: they have more sampling uncertainty and information, lower probability of sampling identical pairs, lower modal probabilities, higher variance, less inequality in their probability distributions, and larger event spaces. If one cares simply about “more vs. less” heterogeneity, then any of these properties will be suitable indices, although we showed that conflicting interpretations can result if this comparison is done across different indices^3^. However, if one is interested in “how much more/less” heterogeneity exists (such as when comparing groups), then only numbers equivalent measures will show appropriate behavior under pooling or decomposition (this was conceptually outlined above, with more rigorous proof in the Supplementary Appendix). The utility of such measures, including their easily understandable units, must be appreciated through real-world applications.

The chief technical obstacle for adopting numbers equivalent measures in psychiatric research is their limitations when applied to non-categorical data. Existing non-categorical numbers equivalent measures satisfy the replication principle^50^, but they still require imposition of a priori stratification on the data, and assumption of a distance metric (see also their idiosyncratic limitations in Supplementary Appendix B and in ref. ^150^). Both limitations preclude adoption in translational psychiatric research. First, if psychiatric science had reliable and valid strata to impose on some data, then we might not have such concern with heterogeneity in the first place. Second, the types of high-dimensional data often used in modern psychiatric research might lie on latent spaces whose geometries do not admit application of predefined distance functions^81^. In such systems, existing non-categorical numbers equivalent measures may fail to accurately measure heterogeneity.

Without a proper measure of heterogeneity, it is impossible to precisely identify the impact of heterogeneity in psychiatric research. That being said, it is trivial to show that heterogeneity is necessary for the occurrence of the Yule-Simpson effect (also known as “Simpson’s Paradox”), which is a straightforward example of implications on effect size estimates. However, it is not clear to what extent this occurs, since one can also show that heterogeneity may be present in the absence of a Yule-Simpson effect. We have also previously recalled that symptomatic heterogeneity is itself a feature of “great imitator” conditions, such as syphilis, and that degree of heterogeneity may be a central feature that differentiates some psychiatric disorders^151^, although in the absence of a proper measure this can only be assumed. To quantify this, a proper measure of heterogeneity is required. Finally, without operationalizing the definition of heterogeneity and understanding its measurable properties, our field will continue to conflate the concept of heterogeneity itself with its causes and consequences, thereby impeding the rigorous study of all three.

Numbers equivalent heterogeneity measures can be relevant for modern translational psychiatric research, but existing indices must be adapted to suit the nature of our data and questions. We must do away with the requirement for a priori data stratification and distance function specification. It will also be interesting to study if, how, and under what circumstances existing measures of meta-analytic heterogeneity and time-series complexity should be expressed in numbers equivalent. Ultimately, development of a rigorous approach for the measurement of heterogeneity will facilitate further studies concerning its causes and consequences in psychiatric research.

## Supplementary information

Supplemental mathematical information

Search query

PDF Version of Supplemental Mathematical information
