# Supplementary material for: The definition and measurement of heterogeneity
Source: Transl Psychiatry. 2020 Aug 24;10:299. doi: 10.1038/s41398-020-00986-0 (PMC7445182; doi:10.1038/s41398-020-00986-0)
Supplement: Supplementary file 2 — Search query [file 41398_2020_986_MOESM2_ESM.pdf]

```
TITLE-ABS-KEY ( psychiatr* )
  AND  KEY ((
      (heterogeneity
       OR  biodiversity
       OR  inequality
       OR  diversity
       OR  (complexity  AND  ( measure  OR  index  OR  coefficient )))
      OR  (
        ( species  AND richness )
         OR  ( hartley  AND entropy )
         OR  ( max  AND entropy ))
      OR  ( shannon  AND entropy )
      OR  ( shannon-wiener  OR  shannon-weaver )
      OR  ( simpson  AND  ( index  OR  concentration ) )
      OR  ( berger-parker )
      OR  ( freeman  AND index )
      OR  ( modvr  OR  avdev )
      OR  ( ( lincoln  OR  petersen )  AND  index )
      OR  ( birthday  AND paradox )
      OR  ( collision  AND entropy )
      OR  ( heip  AND index )
      OR  ( ( herfindahl  OR  herfindahl-hirschman )
      AND  ( index  OR  coefficient ) )
      OR  ( hill  AND numbers )
      OR  ( rÃ©nyi  AND entropy )
      OR  ( tsallis  AND entropy )
      OR  ( lempel-ziv )
      OR  ( huffman  AND code )
      OR  ( statistical  AND evenness )
      OR  ( ( gini  OR  gini-simpson )  AND  ( index  OR  coefficient ) )
      OR  ( lorenz  AND curve )
      OR  ( theil  AND  ( index  OR  coefficient ) )
      OR  ( atkinson  AND  ( index  OR  coefficient ) )
      OR  ( dalton  AND  ( index  OR  coefficient ) )
      OR  ( "generalized entropy index" )
      OR  ( ( pietra  OR  ( robin  AND hood )  OR  schutz )  AND  index )
      OR  ( pielou  AND  evenness )
      OR  ( "functional diversity" )
      OR  ( rao's  AND quadratic  AND entropy )
      OR  ( quadratic  AND entropy )
      OR  ( jaccard  AND  ( index  OR  coefficient ) )
      OR  ( combinatori* )
      OR  ( ( cluster  AND analysis )  OR  clustering )
      OR  ( ( finite  OR  gaussian )  AND  ( mixture  AND model ) )
      OR  ( latent  AND class  AND analysis )
      OR  ( latent  AND class  AND growth  AND analysis )
      OR  ( normative  AND model* )))
  AND  (
    EXCLUDE ( DOCTYPE ,  "ed" )
    OR  EXCLUDE ( DOCTYPE ,  "no" )
    OR  EXCLUDE ( DOCTYPE ,  "er" )
    OR  EXCLUDE ( DOCTYPE ,  "le" )
    OR  EXCLUDE ( DOCTYPE ,  "cr" )
    OR  EXCLUDE ( DOCTYPE ,  "tb" )
    OR  EXCLUDE ( DOCTYPE ,  "Undefined" )
```

) AND (LIMIT-TO (LANGUAGE , "English"))
