# Supplementary material for: The definition and measurement of heterogeneity
Source: Transl Psychiatry. 2020 Aug 24;10:299. doi: 10.1038/s41398-020-00986-0 (PMC7445182; doi:10.1038/s41398-020-00986-0)
Supplement: Supplementary file 3 — PDF Version of Supplemental Mathematical information [file 41398_2020_986_MOESM3_ESM.pdf]

Supplementary Materials for *The Definition and Measurement of Heterogeneity*

Abraham Nunes (nunes@dal.ca), Thomas Trappenberg, and Martin Alda
Dalhousie University, Halifax, Nova Scotia, Canada

A. Mathematical Details

A.1 Proposed Heterogeneity Axioms

AXIOM 1 (NON-NEGATIVITY)
The heterogeneity measure $h(\mathbf{y})$ is strictly positive $\forall\, \mathbf{y} \in \mathcal{Y} \subseteq \mathbb{R}^n_{\geq 0} \setminus \{\emptyset\}$.

AXIOM 2 (NULL EMPTY SET)
The heterogeneity measure $h : \mathcal{Y} \to \mathbb{R}_{\geq 0}$ equals 0 iff $\mathcal{Y} = \emptyset$.

AXIOM 3 (SYMMETRY)
Given an abundance distribution $\mathbf{y} = (y_i)_{i=1}^n \in \mathcal{Y} \subseteq \mathbb{R}^n_{\geq 0}$ and a permutation function $\sigma : \mathbb{Z}_+ \to \mathbb{Z}_+$, the heterogeneity measure $h : \mathcal{Y} \to \mathbb{R}_{\geq 0}$ satisfies

$$h(\{y_1, y_2, \ldots, y_n\}) = h(\{y_{\sigma(1)}, y_{\sigma(2)}, \ldots, y_{\sigma(n)}\}).$$

AXIOM 4 (CONTINUITY AND DIFFERENTIABILITY)
The heterogeneity measure $h : \mathcal{Y} \to \mathbb{R}_{\geq 0}$ is a continuous and differentiable function $\forall\, \mathbf{y} \in \mathcal{Y}$.

AXIOM 5a (EXTENSIVITY OR MONOTONICITY TO SET SIZE)
Given a family of distributions $\mathbf{y}(n) = (y_i)_{i=1}^n$ with a constant level of inequality for all $n \in \mathbb{N}_+$, the heterogeneity measure h must satisfy

$$h(\mathbf{y}(n + \delta)) > h(\mathbf{y}(n)) \;\forall\, \delta \in \mathbb{N}_+$$

AXIOM 5b (NON-EXTENSIVITY OR INVARIANCE TO SET SIZE)
Given a family of distributions $\mathbf{y}(n) = (y_i)_{i=1}^n$ with a constant level of inequality for all $n \in \mathbb{N}_+$, the

Printed by Wolfram Mathematica Student Edition

heterogeneity measure h must satisfy

$$h(\mathbf{y}(n+\delta)) = h(\mathbf{y}(n)) \; \forall\, \delta \in \mathbb{N}_+$$

Remark. Extensivity is typically desirable for heterogeneity measures corresponding to set size. Measures that are specifically focused on *inequality* generally require non-extensivity.

AXIOM 6 (PRINCIPLE OF TRANSFERS)

Given an abundance vector $\mathbf{y} = (y_i)_{i=1}^n$, if we define a new vector $\mathbf{y}'$ by the following transfer of some small amount of abundance ,

$$y'_k = \begin{cases} y_k - \epsilon & k = j \\ y_k + \epsilon & k = i \\ y_k & k \neq i \wedge k \neq j \end{cases}$$

where $y_j > y_i$ then heterogeneity must increase, with a maximal value attained iff $y_i = y_j$.

AXIOM 7 (THE REPLICATION PRINCIPLE)

We are given $n_s \in \mathbb{N}_{\geq 2}$ systems, with respective distributions $\mathbf{y}_i \; \forall\, i \in \{1, 2, \ldots, n_s\}$, whose domains of support are non-overlapping, but whose heterogeneities are equal:

$$h(\mathbf{y}_i) = h(\mathbf{y}_j) \; \forall\, (i, j) \in \{1, 2, \ldots, n_s\}$$

Letting $\overline{\mathbf{y}}$ be the abundance distribution on the pooled n_s systems, the replication principle states that

$$h(\overline{\mathbf{y}}) = n_s\, h(\mathbf{y}_i) \; \forall\, i \in \{1, 2, \ldots, n_s\}.$$

AXIOM 8 (DECOMPOSABILITY)

Given a system $\mathcal{X}$ with abundance distribution p that is a composition of subsystems $\mathcal{X}^{(1)}, \mathcal{X}^{(2)}, \ldots, \mathcal{X}^{(K)}$ with corresponding abundance distributions $\mathbf{y}_1, \mathbf{y}_2, \ldots, \mathbf{y}_n$, then we define the heterogeneity of the composite system—also known as the total heterogeneity or γ-heterogeneity—as $h^\gamma(\overline{\mathbf{y}})$, although for brevity we will simply denote it as h^γ. The component of total heterogeneity due to within-group factors is also known as the α-heterogeneity, and is denoted as h^α. Finally, the component of heterogeneity due to between-group differences is denoted as h^β and is known as the β-heterogeneity. The heterogeneity measure h is decomposable if (Jost, 2007):

A. There exists a deterministic function Ξ such that $h^\gamma = \Xi(h^\alpha, h^\beta)$
B. The within and between-group components are independent: $h^\alpha \perp\!\!\!\perp h^\beta$
C. The within-group heterogeneity is a lower bound on total heterogeneity: $h^\alpha \leq h^\gamma$
D. The within-group and between-group components have the same units

AXIOM 9 (SCALE INVARIANCE)
Given an abundance vector $\mathbf{y} = (y_i)_{i=1}^{n}$ and a positive scalar $k \in \mathbb{R}_+$, $h(k\,\mathbf{y}) = h(\mathbf{y})$

A.2. Numbers Equivalent

Consider a set $\mathcal{X}$ with probability (abundance) distribution $\mathbf{p} = (p_i)_{i=1}^{n_c^{(p)}}$ and heterogeneity

$$\Pi_q[\mathbf{p}] = \left(\sum_{i=1}^{n_c^{(p)}} p_i^q\right)^{\frac{1}{1-q}} \tag{1}$$

Given a second set $\mathcal{X}'$ with a uniform distribution $u_j = 1/n_c^{(u)}\ \forall\, j \in \{1,\ 2,\ \ldots,\ n_c^{(u)}\}$ whose heterogeneity is such that $\Pi_q[\mathbf{p}] = \Pi_q[\mathbf{u}]$, we can easily see that

In[∘]:=
```
TraditionalForm@PowerExpand@ReplaceAll[{u_j → 1/n_c^(u)}][(Σ_{i=1}^{n_c^(p)} p_i^q)^(1/(1-q)) == (Σ_{j=1}^{n_c^(u)} u_j^q)^(1/(1-q))]
```

Out[∘]//TraditionalForm=

$$\left(\sum_{i=1}^{n_c^p} p_i^q\right)^{\frac{1}{1-q}} = nc^{-\frac{(q-1)\,u}{1-q}}$$

In[∘]:=
```
TraditionalForm@FullSimplify[%]
```

Out[∘]//TraditionalForm=

$$n_c^u = \left(\sum_{i=1}^{n_c^p} p_i^q\right)^{\frac{1}{1-q}}$$

which shows that the heterogeneity of $\mathcal{X}$ is the number of partitions $n_c^{(u)}$ in an equally heterogeneous set $\mathcal{X}'$ with uniform abundance distribution.

A.3. Entropies and the Replication Principle

PROPOSITION A1. Entropies derived from Tsallis' form fail to satisfy the replication principle.

Proof. Assume the total number of partitions in a system composed of Msubsystems is $n = \sum_{i=1}^{M} n_i$, where n_i is the number of partitions in the i'th sample. The probability distribution for system i is $\mathbf{p}_i = (p_{i1},\ p_{i2},\ \ldots,\ p_{in_i})$. Recall that the domains of support for $\mathbf{p}_1,\ \mathbf{p}_2,\ \ldots,\ \mathbf{p}_M$ are disjoint. The Tsallis entropy of a single system is

In[∘]:=
```
T_q[p_i] = 1/(q-1) (1 - Σ_{j=1}^{n_i} p_ij^q)
```

Out[∘]=

$$\frac{1 - \sum_{j=1}^{n_i} p_{i,j}^q}{-1 + q}$$

and the Tsallis entropy of the pooled system, whose probability distribution is $\overline{\mathbf{p}}$, is as follows:

In[•]:=

$$T_q[\overline{p}] = \frac{1}{q-1}\left(1-\sum_{k=1}^{n}\overline{p}_k^q\right)$$

Out[•]=

$$\frac{1-\sum_{k=1}^{n}\overline{p}_k^q}{-1+q}$$

Since the domains for each of the M subsystems is disjoint, we have that

$$\mathbf{P}=\begin{pmatrix} p_{11} & p_{12} & \cdots & p_{1n_1} & 0 & 0 & 0 & 0 & \cdots & 0 & 0 & 0 & 0 & \cdots & 0 & 0 & 0 & 0 \\ 0 & 0 & 0 & 0 & p_{21} & p_{22} & \ldots & p_{2\,n_2} & \cdots & \vdots & \vdots & \vdots & \vdots & \cdots & \vdots & \vdots & \vdots & \vdots \\ \vdots & \vdots & \vdots & \vdots & \vdots & \vdots & \vdots & \vdots & \ddots & \vdots & \vdots & \vdots & \vdots & \cdots & \vdots & \vdots & \vdots & \vdots \\ 0 & 0 & 0 & 0 & 0 & 0 & 0 & 0 & \cdots & p_{i1} & p_{i2} & \ldots & p_{in_i} & \cdots & 0 & 0 & 0 & 0 \\ \vdots & \vdots & \vdots & \vdots & \vdots & \vdots & \vdots & \vdots & \cdots & \vdots & \vdots & \vdots & \vdots & \ddots & \vdots & \vdots & \vdots & \vdots \\ 0 & 0 & 0 & 0 & 0 & 0 & 0 & 0 & \cdots & 0 & 0 & 0 & 0 & \cdots & p_{M1} & p_{M2} & \ldots & p_{Mn_M} \end{pmatrix} \quad (2)$$

and

$$\overline{\mathbf{p}}=\frac{1}{M}\left(p_{11}\;\; p_{12}\;\; \cdots\;\; p_{1n_1}\;\; p_{21}\;\; p_{22}\;\; \ldots\;\; p_{2\,n_2}\;\; \cdots\;\; p_{i1}\;\; p_{i2}\;\; \ldots\;\; p_{in_i}\;\; \cdots\;\; p_{M1}\;\; p_{M2}\;\; \ldots\;\; p_{Mn_M}\right) \quad (3)$$

Substituting (3) into the Tsallis entropy yields the following expression:

In[•]:=

$$T_q[\overline{p}] = \text{PowerExpand}\left[T_q[\overline{p}] /. \left\{\sum_{k=1}^{n}\overline{p}_k^q \to \sum_{i=1}^{M}\sum_{j=1}^{n_i}\left(\frac{p_{ij}}{M}\right)^q\right\}\right]$$

Out[•]=

$$\frac{1-\sum_{i=1}^{M}\sum_{j=1}^{n_i} M^{-q}\, p_{i,j}^q}{-1+q}$$

Rearranging terms,

In[•]:=

$$\text{num} = \text{Numerator}[T_q[\overline{p}]] /. \left\{ 1-\sum_{v1\_=l1\_}^{h1}\sum_{v2\_=l2\_}^{h2} r\_\; q\_ /; \text{FreeQ}[r,\, v1] \wedge \text{FreeQ}[r,\, v2] :\!\!\to 1-r\sum_{v1=l1}^{h1}\sum_{v2=l2}^{h2} q \right\}$$

$$T_q[\overline{p}] = \frac{\text{num}}{\text{Denominator}[T_q[\overline{p}]]};$$

Out[•]=

$$1-M^{-q}\sum_{i=1}^{M}\sum_{j=1}^{n_i} p_{i,j}^q$$

we recall that $\sum_{j=1}^{n_i} p_{i,j}^q = \sum_{j=1}^{n_k} p_{k,j}^q$ for all i, k. Letting $\lambda_i = \sum_{j=1}^{n_i} p_{i,j}^q$, $T_q[\overline{\mathbf{p}}]$ simplifies to

In[•]:=

$$T_q[\overline{p}] = \text{ReplaceAll}\left[\left\{\sum_{i=1}^{M}\sum_{j=1}^{n_i} p_{i,j}^q \to M\,\lambda_i\right\}\right][T_q[\overline{p}]]$$

Out[•]=

$$\frac{1-M^{1-q}\,\lambda_i}{-1+q}$$

and $T_q[\mathbf{p}_i]$ simplifies to the following.

In[∘]:=
```
Tq[pi] = ReplaceAll[{∑_{j=1}^{ni} p_ij^q → λi}][Tq[pi]]
```

Out[∘]= $\frac{1-\lambda_i}{-1+q}$

The replication principle states that the following equality must hold: $T_q[\mathbf{p}] = M\, T_q[\mathbf{p}_i]$. We conclude the proof by showing that it does not. Note that $\lambda_i = \sum_{j=1}^{n_i} p_{i,j}^q$.

In[∘]:=
```
pa1eq = ApplySides[ExpandAll[#×(q-1)]&, Tq[p̄] == M Tq[pi]]
```

Out[∘]= $1 - M^{1-q}\lambda_i == M - M\lambda_i$

In[∘]:=
```
pa1eq = ApplySides[(#-1)/(M-M^(1-q))&,FullSimplify@pa1eq]
```

Out[∘]= $\lambda_i == \frac{-1+M}{M-M^{1-q}}$

This equality holds only at $q = 1$, which is irrelevant since $T_q[\mathbf{p}]$ is undefined at $q = 1$ (we will prove this in the limiting case of $q \to 1$ below). Showing that the above equality is not generally true is easy under a counterexample. Substituting $\lambda_i \to (p_{in_i} - \epsilon)^q + (p_{in_i-1} + \epsilon)^q + \sum_{j=1}^{n_i-2} p_{ij}^q$ and differentiating with respect to ϵ, we obtain our result.

In[∘]:=
```
pa1eq = pa1eq /. λi → (p_{i ni} - ϵ)^q + (p_{i ni-1} + ϵ)^q + ∑_{j=1}^{ni-2} p_ij^q
```

Out[∘]= $(\epsilon + p_{i,-1+n_i})^q + (-\epsilon + p_{i,n_i})^q + \sum_{j=1}^{-2+n_i} p_{i,j}^q == \frac{-1+M}{M-M^{1-q}}$

In[∘]:=
```
pa1eq = ApplySides[D[#, M]&, pa1eq]
```

Out[∘]= $0 == \frac{1}{M-M^{1-q}} - \frac{(-1+M)\left(1-M^{-q}(1-q)\right)}{\left(M-M^{1-q}\right)^2}$

In[∘]:=
```
pa1eq = ExpandAll@ApplySides[
    (#+ ((-1+M) (1-M^-q (1-q)))/(M-M^(1-q))^2) (M-M^(1-q))^2& , pa1eq]
```

Out[∘]= $-1 + M - M^{1-q} + M^{-q} + M^{1-q}q - M^{-q}q == M - M^{1-q}$

In[∘]:=
```
pa1eq = ApplySides[# -M+M^(1-q)+M^-q q-M^-q &, pa1eq]
```

Out[∘]= $-1 + M^{1-q}q == -M^{-q} + M^{-q}q$

In[ ]:=
```
pa1eq = ApplySides[#/(-1+M^q+q-M q) & , FullSimplify@pa1eq]
```

Out[ ]= $M^{-q} == 0$

In[ ]:=
```
FullSimplify[pa1eq, Assumptions→{M≥1, q>0}]
```

Out[ ]= False

Thus, for $q \neq 1$, the Tsallis family of entropies does not satisfy the replication principle. We now show the $q \to 1$ case. We use L'Hopital's rule to obtain $T_1[\mathbf{p}]$:

In[ ]:=
$$T_1[p_i] = \text{Limit}\left[\frac{D\left[1-\sum_{j=1}^{n_i} p_{ij}^q,\ q\right]}{D[q-1,\ q]},\ q\to 1\right]\ /.\ \lim_{e\_\to a\_} -\sum_{v\_=l\_}^{u} \text{Log}[x\_]\ x\_^{e\_} :\to -\sum_{v=l}^{u} \lim_{e\to a} (\text{Log}[x]\ x^e)$$

Out[ ]= $$-\sum_{j=1}^{n_i} \text{Log}[p_{i,j}]\ p_{i,j}$$

and similarly for $T_1[\overline{\mathbf{p}}]$

In[ ]:=
$$T_1[\overline{p}] = \text{Limit}\left[\frac{D\left[1-\sum_{i=1}^{M}\sum_{j=1}^{n_i}\left(\frac{p_{ij}}{M}\right)^q,\ q\right]}{D[q-1,\ q]},\ q\to 1\right]\ /.\left\{\lim_{e\_\to a\_} -\sum_{v1\_=l1\_}^{u1}\sum_{v\_=l\_}^{u} \text{Log}[x\_]\ x\_^{e\_} :\to -\sum_{v1=l1}^{u1}\sum_{v=l}^{u} \lim_{e\to a}(\text{Log}[x]\ x^e)\right\}$$

Out[ ]= $$-\sum_{i=1}^{M}\sum_{j=1}^{n_i} \frac{\text{Log}\left[\frac{p_{i,j}}{M}\right] p_{i,j}}{M}$$

In[ ]:=
$$T_1[\overline{p}] = T_1[\overline{p}]\ /.\ \left\{-\sum_{i=1}^{M}\sum_{j=1}^{n_i}\frac{\text{Log}\left[\frac{p_{i,j}}{M}\right]\ p_{i,j}}{M} \to -\frac{1}{M}\sum_{i=1}^{M}\sum_{j=1}^{n_i}\text{Log}\left[\frac{p_{i,j}}{M}\right]\ p_{i,j}\right\}$$

Out[ ]= $$-\frac{\sum_{i=1}^{M}\sum_{j=1}^{n_i}\text{Log}\left[\frac{p_{i,j}}{M}\right] p_{i,j}}{M}$$

In[ ]:=
$$\text{pa1eq2} = M\ T_1[\overline{p}] == M^2\ T_1[p_i]$$

Out[ ]= $$-\sum_{i=1}^{M}\sum_{j=1}^{n_i}\text{Log}\left[\frac{p_{i,j}}{M}\right] p_{i,j} == -M^2\sum_{j=1}^{n_i}\text{Log}[p_{i,j}]\ p_{i,j}$$

In[ ]:=
$$\text{pa1eq2} = \text{pa1eq2}\ /.\ -\sum_{i=1}^{M}\sum_{j=1}^{n_i}\text{Log}\left[\frac{p_{i,j}}{M}\right]\ p_{i,j} \to -\sum_{i=1}^{M}\sum_{j=1}^{n_i} p_{i,j}\text{Log}[p_{i,j}] - \text{Log}[M]\sum_{i=1}^{M}\sum_{j=1}^{n_i} p_{i,j}$$

Out[ ]= $$-\text{Log}[M]\sum_{i=1}^{M}\sum_{j=1}^{n_i} p_{i,j} - \sum_{i=1}^{M}\sum_{j=1}^{n_i}\text{Log}[p_{i,j}]\ p_{i,j} == -M^2\sum_{j=1}^{n_i}\text{Log}[p_{i,j}]\ p_{i,j}$$

Since $\sum_{i=1}^{M}\sum_{j=1}^{n_i} p_{i,j} = M$ and letting $\sum_{j=1}^{n_i}\text{Log}[p_{i,j}]\,p_{i,j} = \lambda_i$,

In[∘]:=
```
pa1eq2 = pa1eq2 /. {
```
$$\sum_{i=1}^{M}\sum_{j=1}^{n_i} p_{i,j} \to M,$$
$$\sum_{j=1}^{n_i} \mathrm{Log}[p_{i,j}]\ p_{i,j} \to \lambda_i,$$
$$\sum_{i=1}^{M}\sum_{j=1}^{n_i} \mathrm{Log}[p_{i,j}]\ p_{i,j} \to M\ \lambda_i$$
```
}
```

Out[∘]= $-M\,\mathrm{Log}[M] - M\,\lambda_i == -M^2\,\lambda_i$

In[∘]:=
```
Flatten@Solve[ApplySides[FullSimplify[#/M]&,pa1eq2], λi]
```

Out[∘]= $\left\{\lambda_i \to \frac{\mathrm{Log}[M]}{-1+M}\right\}$

Since the LHS is variable and the RHS is constant, this equality cannot hold.

□

A.4. Properties of the Rényi Heterogeneity

The Rényi heterogeneity is defined as

In[∘]:= $\Pi_q[p] = \left(\sum_{i=1}^{m} p_i^q\right)^{\frac{1}{1-q}};$

In[∘]:= $\Pi_0[p] = \sum_{i=1}^{m} \delta_{p_i>0};$

In[∘]:= $\Pi_1[p] = e^{-\sum_{i=1}^{m} p_i \mathrm{Log}[p_i]};$

In[∘]:= $\Pi_\infty[p] = \frac{1}{\mathrm{Max}[p]};$

PROPOSITION A2. The Rényi heterogeneity is non-negative.

The proof is trivial since $p_i \geq 0\ \forall\, i \in \{1, 2, \ldots, n\}$.

PROPOSITION A3. The Rényi heterogeneity is symmetric.

The proof is trivial by the commutative properties of addition, maximum, and the indicator function.

PROPOSITION A4. The Rényi heterogeneity is continuous and differentiable with respect to the probability distribution **p**.

Proof. The Rényi heterogeneity can be viewed as a composition of continuous functions, and is therefore continuous for $0 < p_i \leq 1$. At $q \neq 1$:

In[∘]:=
```
f[x_] := x_i^q
g[x_, v_:i,l_:1,u_:m] := Sum[x, {v, 1, u}]
h[x_] := x^(1/(1-q))
h@g@f[p]
```

Out[∘]= $\left(\sum_{i=1}^{m} p_i^q\right)^{\frac{1}{1-q}}$

As $q \to 1$, we have the following:

In[∘]:=
```
I[x_]:= -Log[x_i]
E[x_, v_:i,l_:1,u_:m][y_]:=Sum[x_i y, {v, 1, u}]
Exp@E[p]@I[p]
```

Out[∘]= $e^{\sum_{i=1}^{m} -\mathrm{Log}[p_i]\, p_i}$

The derivative of the Rényi heterogeneity in terms of it’s component functions is

In[∘]:=
```
ClearAll[f,g,h]
D[h@g@f[x], x]
```

Out[∘]= f′[x] g′[f[x]] h′[g[f[x]]]

We can verify that the component derivatives exist so long as $q \neq 1$,

In[∘]:=
```
D[g[x]^(1/(1-q)), g[x]]//FullSimplify
```

Out[∘]= $\dfrac{g[x]^{-\frac{q}{-1+q}}}{1-q}$

In[∘]:=
```
FullSimplify[D[Sum[f[x_i], {i, 1, m}], f[x_i]], Assumptions→{1≤i≤m, i∈Integers}]
```

Out[∘]= 1

In[∘]:=
```
FullSimplify[D[f[x_i], x_i], Assumptions→{1≤i≤m}]
```

Out[∘]= f′[x_i]

although one could easily show that the derivatives exist for the $q \to 1$ limit. The derivative of Rényi heterogeneity with respect to p_k therefore exists by the chain rule.

$$\frac{\partial \Pi_q}{\partial p_k} = \frac{\partial \Pi_q}{\partial h} \frac{\partial h}{\partial g} \frac{\partial g}{\partial f} \frac{\partial f}{\partial p_k} \tag{4}$$

□

PROPOSITION A5. The Rényi heterogeneity is monotonic to set size.

Proof. For $\mathbf{p} = (p_i)_{i=1}^n$, recall that $\Pi_0[\mathbf{p}] = n$. One can show that the derivative of the Rényi entropy with respect to q is proportional to the negative Kullback-Leibler divergence $\sum_{i=1}^m z_i \,\text{Log}[z_i/p_i]$, where $z_i = p_i^q / \sum_{i=1}^m p_i^q$:

In[∘]:=
```
pa5expr = -D[Log[Π_q[p]], q]
```

Out[∘]=
$$-\frac{\text{Log}\left[\sum_{i=1}^m p_i^q\right]}{(1-q)^2} - \frac{\sum_{i=1}^m \text{Log}[p_i]\, p_i^q}{(1-q)\sum_{i=1}^m p_i^q}$$

Letting $z_i = p_i^q / \sum_{i=1}^m p_i^q$ and substituting, we see that

In[∘]:=
```
pa5expr = pa5expr/.{
    (Σ_{i=1}^m Log[p_i] p_i^q)/((1-q) Σ_{i=1}^m p_i^q) → (Σ_{i=1}^m z_i Log[p_i])/(1-q)}
```

Out[∘]=
$$-\frac{\text{Log}\left[\sum_{i=1}^m p_i^q\right]}{(1-q)^2} - \frac{\sum_{i=1}^m \text{Log}[p_i]\, z_i}{1-q}$$

In[∘]:=
```
pa5expr = PowerExpand@Together@pa5expr /. {
    Log[Σ_{i=1}^m p_i^q] → q Σ_{i=1}^m z_i Log[p_i] - Σ_{i=1}^m z_i Log[z_i]}/. {
    -Σ_{i=1}^m Log[p_i] z_i + Σ_{i=1}^m Log[z_i] z_i → Σ_{i=1}^m z_i Log[z_i/p_i]}
```

Out[∘]=
$$\frac{\sum_{i=1}^m \text{Log}\left[\frac{z_i}{p_i}\right] z_i}{(-1+q)^2}$$

This means that $\Pi_q[\mathbf{p}]$ is nondecreasing with respect to q, and thus $(\Pi_q[\mathbf{p}]/\Pi_0[\mathbf{p}]) \leq 1$. Now define a family of distributions $\mathbf{p}(n) = (p_i)_{i=1}^n$ with a constant level of evenness, $\Pi_q[\mathbf{p}(n)]/\Pi_0[\mathbf{p}(n)]$. Therefore,

In[∘]:=
```
pa5eq = Π_q[p[n]]/Π_0[p[n]] == Π_q[p[n+1]]/Π_0[p[n+1]]
```

Out[∘]=
$$\frac{\Pi_q[p[n]]}{\Pi_0[p[n]]} == \frac{\Pi_q[p[1+n]]}{\Pi_0[p[1+n]]}$$

In[∘]:=
```
pa5eq = pa5eq/.{Π_0[p[n]]→n, Π_0[p[n+1]]→n+1}
```

Out[∘]=
$$\frac{\Pi_q[p[n]]}{n} == \frac{\Pi_q[p[1+n]]}{1+n}$$

In[∘]:=
```
ApplySides[#/Πq[p[n]]&, ApplySides[# (n+1) &, pa5eq]]
```

Out[∘]=
$$\frac{1+n}{n} == \frac{\Pi_q[p[1+n]]}{\Pi_q[p[n]]}$$

□

PROPOSITION A6. The Rényi heterogeneity obeys the principle of transfers.

Proof. For some small ϵ transfer from p_n to p_{n-1}, when before the transfer $p_n > p_{n-1}$, the Rényi heterogeneity is

In[∘]:=
$$\Pi_q[p'] = \left((p_{n-1}+\epsilon)^q + (p_n-\epsilon)^q + \sum_{i=1}^{n-2} p_i^q\right)^{\frac{1}{1-q}};$$

Differentiating with respect to ϵ and solving for $\partial_\epsilon \Pi_q[\mathbf{p}']$ gives

In[∘]:=
```
ϵ* = Extract[Solve[D[Πq[p′], ϵ]==0, ϵ], {1, 1, 2}]
```

Out[∘]=
$$\frac{1}{2}(-p_{-1+n}+p_n)$$

which is a transfer that would set $p_n = p_{n-1}$. We can show that $\Pi_q[\mathbf{p}']$ is maximized at ϵ_* by demonstrating that $\Pi_q[\mathbf{p}']$ has constant negative curvature.

In[∘]:=
```
pa6curv = FullSimplify[D[Πq[p′], {ϵ, 2}]/.ϵ→ϵ*];
pa6ineq = pa6curv ≤ 0
```

Out[∘]=
$$-\frac{8\,q\,(p_{-1+n}+p_n)^{-2+q}\left(2^{1-q}(p_{-1+n}+p_n)^q+\sum_{i=1}^{-2+n} p_i^q\right)^{\frac{1}{1-q}}}{2\,(p_{-1+n}+p_n)^q+2^q\sum_{i=1}^{-2+n} p_i^q} \le 0$$

In[∘]:=
```
pa6ineq = ApplySides[# Denominator[pa6curv]&, pa6ineq];
pa6ineq = ApplySides[# / Extract[pa6ineq, {1,4}]&, pa6ineq]
```

Out[∘]=
$$-8\,q\,(p_{-1+n}+p_n)^q \le 0$$

Since $(p_{-1+n}+p_n) > 0$ and $q > 0$, the inequality is true.

□

PROPOSITION A7. The Rényi heterogeneity obeys the replication principle.

Proof. The Rényi heterogeneity for a single subsystem is

In[∘]:=
$$\Pi_q[p_i] = \left(\sum_{j=1}^{n_i} p_{ij}^q\right)^{\frac{1}{1-q}};$$

and for the pooled system is

In[ ]:=
$$\Pi_q[\overline{p}] = \left(\sum_{i=1}^{M}\sum_{j=1}^{n_i}\left(\frac{p_{ij}}{M}\right)^q\right)^{\frac{1}{1-q}};$$

The replication principle states that

In[ ]:=
```
pa7eq = Πq[p̄] == M Πq[pi]
```

Out[ ]=
$$\left(\sum_{i=1}^{M}\sum_{j=1}^{n_i}\left(\frac{p_{i,j}}{M}\right)^q\right)^{\frac{1}{1-q}} == M\left(\sum_{j=1}^{n_i} p_{i,j}^q\right)^{\frac{1}{1-q}}$$

In[ ]:=
```
pa7eq = ApplySides[PowerExpand, pa7eq] /.{
```
$$\left(\sum_{i=1}^{M}\sum_{j=1}^{n_i} M^{-q}\; p_{i,j}^q\right)^{\frac{1}{1-q}} \to \left(M^{-q}\sum_{i=1}^{M}\sum_{j=1}^{n_i} p_{i,j}^q\right)^{\frac{1}{1-q}}\}$$

Out[ ]=
$$\left(M^{-q}\sum_{i=1}^{M}\sum_{j=1}^{n_i} p_{i,j}^q\right)^{\frac{1}{1-q}} == M\left(\sum_{j=1}^{n_i} p_{i,j}^q\right)^{\frac{1}{1-q}}$$

Letting $\lambda_i = \sum_{j=1}^{n_i} p_{i,j}^q$:

In[ ]:=
```
pa7eq = pa7eq /.{
```
$$\sum_{j=1}^{n_i} p_{i,j}^q \to \lambda_i\}$$

Out[ ]=
$$\left(M^{-q}\sum_{i=1}^{M}\sum_{j=1}^{n_i} p_{i,j}^q\right)^{\frac{1}{1-q}} == M\,\lambda_i^{\frac{1}{1-q}}$$

Finally, since $\lambda_i = \lambda_k\ \forall\ i,\ k \in \{1, 2, \ldots, M\}$,

In[ ]:=
```
pa7eq = pa7eq /.{
```
$$\left(M^{-q}\sum_{i=1}^{M}\lambda_i\right)^{\frac{1}{1-q}} \to \left(M^{1-q}\lambda_i\right)^{\frac{1}{1-q}}\}$$

Out[ ]=
$$\left(M^{-q}\sum_{i=1}^{M}\sum_{j=1}^{n_i} p_{i,j}^q\right)^{\frac{1}{1-q}} == M\,\lambda_i^{\frac{1}{1-q}}$$

In[ ]:=
```
PowerExpand@pa7eq
```

Out[ ]=
$$M^{-\frac{q}{1-q}}\left(\sum_{i=1}^{M}\sum_{j=1}^{n_i} p_{i,j}^q\right)^{\frac{1}{1-q}} == M\,\lambda_i^{\frac{1}{1-q}}$$

□

PROPOSITION A8. The Rényi heterogeneity has a multiplicative decomposition.

The proof can be found in Jost (2007).

PROPOSITION A9. The Rényi heterogeneity is scale invariant.

The proof is trivial since the Rényi heterogeneity operates on probability distributions.

A.5. Shannon Entropy and Typical Set Size

Given a total of n observations from a system with m classes, let the observed abundance distribution over classes be denoted $\mathbf{y} = (y_1, y_2, \ldots, y_m)$. The effective number of typical such samples of size n is

In[ ]:=
```
S = n! / Product[(p_i!), {i, 1, m}]
```

Out[ ]=
$$\frac{n!}{\prod_{i=1}^{m} p_i!}$$

Taking logs of both sides, we have the following.

In[ ]:=
```
logS = PowerExpand@Log[S] /.Log[Product[x_, {v_, 1_, u_}]] :> Sum[Log[x], {v, 1, u}]
```

Out[ ]=
$$\mathrm{Log}[n!] - \sum_{i=1}^{m} \mathrm{Log}[p_i!]$$

Now we can employ Stirling's approximation:

In[ ]:=
```
stirlingapprox[x_] = {Log[x!]→ x Log[x]-x};
logS = logS /. Flatten[{stirlingapprox[n], stirlingapprox[p_i]}];
logS = logS /. Sum[(-a_ + b_), {v_, 1_, u_}] :> -Sum[a, {v, 1, u}] + Sum[b, {v, 1, u}]
```

Out[ ]=
$$-n + n\,\mathrm{Log}[n] + \sum_{i=1}^{m} p_i - \sum_{i=1}^{m} \mathrm{Log}[p_i]\,p_i$$

In[ ]:=
```
logS = logS/. Sum[p_i, {i, 1, m}]→n /.n Log[n]→ Sum[p_i, {i, 1, m}] Log[n]
```

Out[ ]=
$$\sum_{i=1}^{m} \mathrm{Log}[n]\,p_i - \sum_{i=1}^{m} \mathrm{Log}[p_i]\,p_i$$

In[ ]:=
```
logS = logS/. Sum[Log[b_] a_, {v_, 1_, u_}] - Sum[Log[a_] a_, {v_, 1_, u_}] :> -Sum[a Log[a/b], {i, 1, u}]
```

Out[ ]=
$$-\sum_{i=1}^{m} \mathrm{Log}\left[\frac{p_i}{n}\right] p_i$$

When $n = 1$, we have the Shannon Entropy, whose exponential is the Rényi heterogeneity of order 1.

In[ ]:=
```
Exp[logS/.n→1]
```

Out[ ]=
$$e^{-\sum_{i=1}^{m} \mathrm{Log}[p_i]\,p_i}$$

B. Non-Categorical Heterogeneity Measures

B.1. Rao’s Quadratic Entropy & Direct Variants

THe generalized Rao’s Quadratic entropy (RQE) originally presented by Chiu & Chao (2014), is

In[ ]:=
$$Q_q = \sum_{i=1}^{n}\sum_{j=1}^{n} d_{ij}\,(p_i p_j)^q;$$

At q=0, this becomes the functional attribute diversity (FAD) index (Walker et al. 1999):

In[ ]:=
```
FAD = Qq/.q→0
```

Out[ ]=
$$\sum_{i=1}^{n}\sum_{j=1}^{n} d_{i,j}$$

which is simply the sum of all pairwise distances between classes (i.e. sum of all elements in *D*). Like the observed richness measure (Equation 9 in the main text) the FAD treats all classes as equally important, regardless of their abundance. At q=1, we have the original RQE (Rao, 1982):

In[ ]:=
```
Q1 = Qq/.q→1
```

Out[ ]=
$$\sum_{i=1}^{n}\sum_{j=1}^{n} p_i\, p_j\, d_{i,j}$$

which is the arithmetic average pairwise distance between classes. At q=1, the distance between a pair of classes is weighted in proportion to the probability that the pair will be sampled.

B.2. Numbers Equivalent RQE

When we set $d_{ij} = (1 - \delta_{ij})$, where δ_{ij} is Kronecker’s delta, then RQE becomes the Gini-Simpson index:

In[ ]:=
```
gsi = Q1/.dij→(1-δij)
```

Out[ ]=
$$\sum_{i=1}^{n}\sum_{j=1}^{n} p_i\, p_j\, (1 - \delta_{i,j})$$

In[ ]:=
$$\text{gsi} = \text{gsi} \;/.\left\{\sum_{i=1}^{n}\sum_{j=1}^{n} p_i\; p_j\; (1-\delta_{i,j}) \rightarrow \sum_{i=1}^{n}\sum_{j=1}^{n} p_i\; p_j - \sum_{i=1}^{n}\sum_{j=1}^{n} p_i\; p_j\; \delta_{i,j}\right\}$$

Out[ ]=
$$\sum_{i=1}^{n}\sum_{j=1}^{n} p_i\, p_j - \sum_{i=1}^{n}\sum_{j=1}^{n} p_i\, p_j\, \delta_{i,j}$$

In[ ]:=
```
gsi = gsi /.{
```
$$\sum_{i=1}^{n}\sum_{j=1}^{n} p_i\ p_j \to 1,$$
$$\sum_{i=1}^{n}\sum_{j=1}^{n} p_i\ p_j\ \delta_{i,j} \to \sum_{i=1}^{n} p_i^2\}$$

Out[ ]=
$$1 - \sum_{i=1}^{n} p_i^2$$

Ricotta & Szeidl (2009) exploited this fact in order to express RQE in numbers equivalent (which can then satisfy the replication principle). Their transformation is as follows. The Rényi heterogeneity at q=2, the inverse Simpson concentration index, can be expressed as the following function of the GSI:

In[ ]:=
$$S_{inv} = \frac{1}{1-gsi}$$

Out[ ]=
$$\frac{1}{\sum_{i=1}^{n} p_i^2}$$

Since GSI $= \sum_{i=1}^{n}\sum_{j=1}^{n}(1-\delta_{ij})\, p_i\, p_j$, we have that

In[ ]:=
$$S_{inv} = S_{inv} /. \sum_{i=1}^{n} p_i^2 \to 1 - \sum_{i=1}^{n}\sum_{j=1}^{n} p_i\ p_j\ (1-\delta_{i,j})$$

Out[ ]=
$$\frac{1}{1-\sum_{i=1}^{n}\sum_{j=1}^{n} p_i\ p_j\ (1-\delta_{i,j})}$$

At this point, we replace the categorical distance $1-\delta_{ij}$ with a distance matrix whose values are all scaled to [0, 1].

In[ ]:=
$$S_{inv} = S_{inv} /. (1-\delta_{i,j}) \to \frac{d_{ij}-\text{min}[d]}{\text{max}[d]-\text{min}[d]}$$

Out[ ]=
$$\frac{1}{1-\sum_{i=1}^{n}\sum_{j=1}^{n} \frac{p_i\ p_j\ (-\text{min}[d]+d_{i,j})}{\text{max}[d]-\text{min}[d]}}$$

In[ ]:=
$$S_{inv} = S_{inv} /. \sum_{i=1}^{n}\sum_{j=1}^{n} \frac{p_i\ p_j\ (-\text{min}[d]+d_{i,j})}{\text{max}[d]-\text{min}[d]} \to \frac{\left(-\sum_{i=1}^{n}\sum_{j=1}^{n} p_i\ p_j\ \text{min}[d]+\sum_{i=1}^{n}\sum_{j=1}^{n} p_i\ p_j\ d_{i,j}\right)}{\text{max}[d]-\text{min}[d]}$$

Out[ ]=
$$\frac{1}{1-\frac{-\sum_{i=1}^{n}\sum_{j=1}^{n}\text{min}[d]\ p_i\ p_j+\sum_{i=1}^{n}\sum_{j=1}^{n} p_i\ p_j\ d_{i,j}}{\text{max}[d]-\text{min}[d]}}$$

In[ ]:=
```
S_inv = S_inv /.{
    Sum[Sum[p_i p_j d_{i,j}, {j,1,n}], {i,1,n}] → HoldForm[Q_1],
    Sum[Sum[min[d] p_i p_j, {j,1,n}], {i,1,n}] → min[d]
    }
```

Out[ ]=
$$\frac{1}{1-\frac{Q_1-\min[d]}{\max[d]-\min[d]}}$$

The resulting numbers equivalent RQE index $\hat{Q}_e$ is

In[ ]:=
```
Q̂e = FullSimplify[S_inv]
```

Out[ ]=
$$\frac{-\max[d]+\min[d]}{Q_1-\max[d]}$$

Unfortunately, this formula appears useful only for ultrametric distances, which is an assumption not generally met by most datasets. When distances are not ultrametric, $\hat{Q}_e$ may yield results that are unintuitive and difficult to interpret (Chao, Chiu, & Jost, 2014).

For example, consider the parameterized distance matrix.

In[ ]:=
$$\text{dmtx}[h\_] = \begin{pmatrix} 0 & 1 & \sqrt{\frac{1}{4}+h^2} \\ 1 & 0 & \sqrt{\frac{1}{4}+h^2} \\ \sqrt{\frac{1}{4}+h^2} & \sqrt{\frac{1}{4}+h^2} & 0 \end{pmatrix};$$

and probability distribution,

In[ ]:=
$$\text{prob}[\theta\_] := \left\{\frac{1}{3}, \frac{1}{3}+\theta, \frac{1}{3}-\theta\right\}$$

which gives the following formula for the numbers equivalent RQE.

In[ ]:=
$$\text{NeqRQEultra}[h\_, \theta\_] := \frac{1}{1-\text{prob}[\theta].\left(\frac{\text{dmtx}[h]-\text{Min}[\text{dmtx}[h]]}{\text{Max}[\text{dmtx}[h]]-\text{Min}[\text{dmtx}[h]]}\right).\text{prob}[\theta]}$$

The distance matrix above shows the pairwise distances between the vertices of a triangle. We demonstrate this graphically here. This shows that the distance between three points is ultrametric when those points form an isosceles triangle.

Out[ ]=

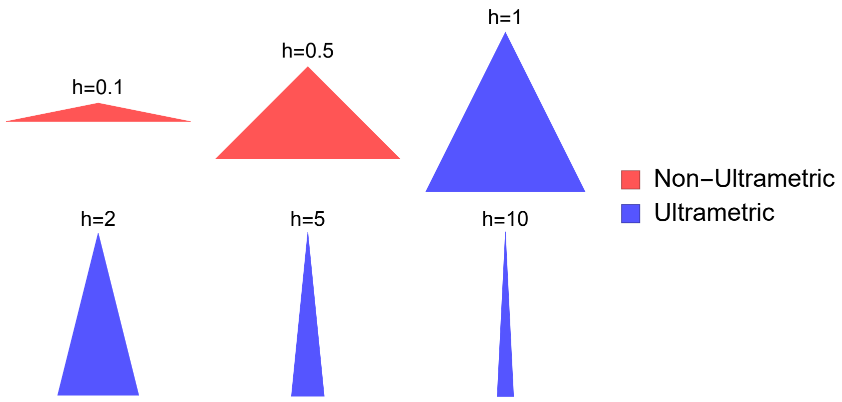

One problem that this causes with the numbers equivalent RQE is that heterogeneity increases with *h* when the distance function is *not* ultrametric, but the relationship changes when the distance function becomes ultrametric. We show this in the plot below.

Out[ ]=

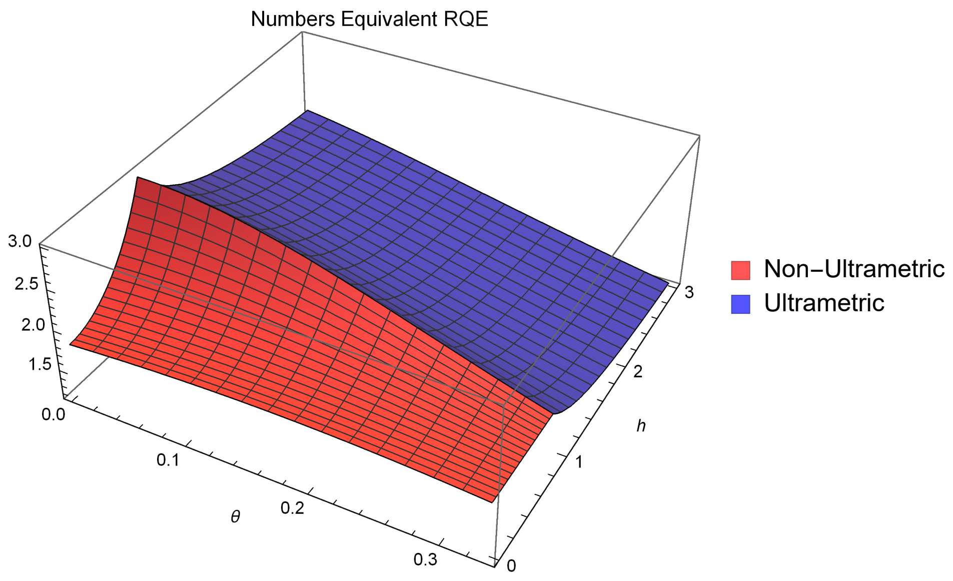

It is left as an exercise to show that the numbers equivalent RQE in this case is maximized at the transition point from an ultrametric to a non-ultrametric distance, which here is at $h = \sqrt{3/4}$.

B.3. Functional Hill Numbers

Chiu & Chao (2014) introduced the functional Hill numbers of order q , for which they first presented a generalized RQE which they then normalize by Q_1:

In[ ]:=

$$F_q = \left(\frac{Q_q}{Q_1}\right)^{\frac{1}{2(1-q)}}$$

Out[ ]=

$$\left(\frac{\sum_{i=1}^{n} \sum_{j=1}^{n} (p_i\, p_j)^q\, d_{i,j}}{\sum_{i=1}^{n} \sum_{j=1}^{n} p_i\, p_j\, d_{i,j}}\right)^{\frac{1}{2(1-q)}}$$

Their derivation is an enjoyable exercise worth reading for those interested. Unfortunately, when the abundance distribution is perfectly even, their measure always yields the observed richness *n*, which is easy to prove directly:

In[ ]:=
```
b3expr = F_q /. {p_i → 1/n, p_j → 1/n}
```

Out[ ]=
$$\left(\frac{\sum_{i=1}^{n}\sum_{j=1}^{n}\left(\frac{1}{n^2}\right)^q d_{i,j}}{\sum_{i=1}^{n}\sum_{j=1}^{n}\frac{d_{i,j}}{n^2}}\right)^{\frac{1}{2(1-q)}}$$

In[ ]:=
```
b3expr = b3expr /. {(Σ_{i=1}^n Σ_{j=1}^n (1/n^2)^q d_{i,j}) / (Σ_{i=1}^n Σ_{j=1}^n d_{i,j}/n^2) → n^(2(1-q)) (Σ_{i=1}^n Σ_{j=1}^n d_{i,j}) / (Σ_{i=1}^n Σ_{j=1}^n d_{i,j})}
PowerExpand@b3expr
```

Out[ ]=
$$\left(n^{2(1-q)}\right)^{\frac{1}{2(1-q)}}$$

Out[ ]= n

□

The above problem, and one other, can be observed in the following example. Consider a parameterized distance matrix

In[ ]:=
```
dmtx[h]//MatrixForm
```

Out[ ]//MatrixForm=
$$\begin{pmatrix} 0 & 1 & \sqrt{\frac{1}{4}+h^2} \\ 1 & 0 & \sqrt{\frac{1}{4}+h^2} \\ \sqrt{\frac{1}{4}+h^2} & \sqrt{\frac{1}{4}+h^2} & 0 \end{pmatrix}$$

and probability distribution,

In[ ]:=
```
prob[θ]
```

Out[ ]=
$$\left\{\frac{1}{3}, \frac{1}{3}+\theta, \frac{1}{3}-\theta\right\}$$

which gives the following formula for the functional Hill numbers.

In[ ]:=
```
pp[θ_]:=prob[θ]⊗prob[θ]
dpp[h_, θ_]:=dmtx[h]×pp[θ]
FuncHillultra[q_][h_, θ_]:= { (Total[Total[dmtx[h] (prob[θ]⊗prob[θ])^q]] / prob[θ].dmtx[h].prob[θ])^(1/(2(1-q)))   q≠1
                              e^(-Total[Total[dpp[h,θ]/(2Total[Total[dpp[h,θ]]]) Log[pp[θ]]]])                  q==1
```

We can use this to observe some properties of F_q graphically (here we use the $q = 1$ setting).

Out[ ]=

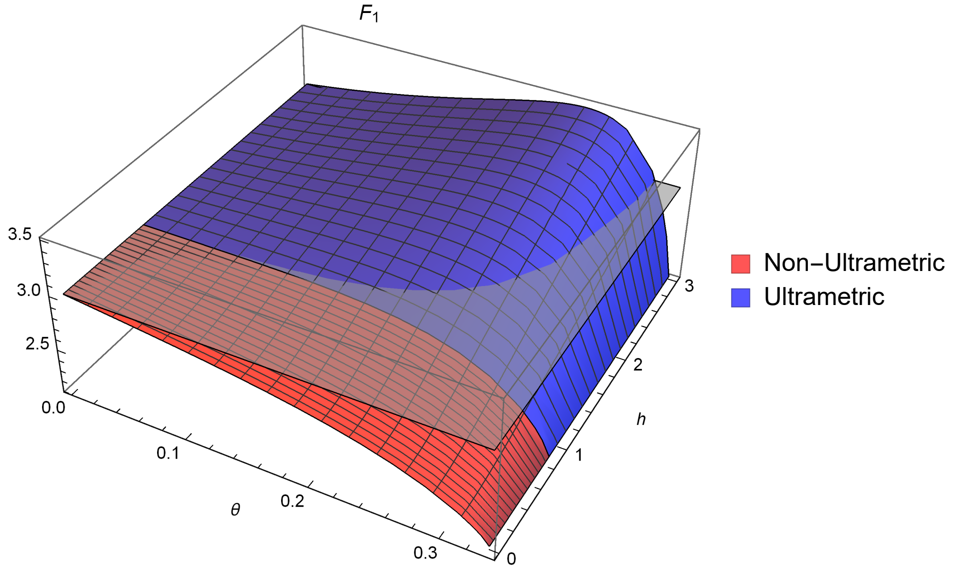

We observe the property in which $F_q = 3$ whenever all probabilities are equal (here 1/3). Another concern is that under the ultrametric regime, the F_q first *increases* with progressively greater inequality in the abundance distribution, before decreasing again at the extremes of inequality. However, in the non-ultrametric region, the gradient is steepest with respect to the abundance distribution, rather than the distance metric. Therefore, F_q shows different behaviour under non-ultrametric and ultrametric distance functions, and in general may be more sensitive to abundance inequality than dissimilarity. This could be of a particular concern when the categories over which abundance is measured are ill-defined or unreliable.

B.4. The Leinster-Cobbold Index

The Leinster-Cobbold index L_q is defined as

In[ ]:=

$$L_q = \left(\sum_{i=1}^{n} p_i^q \left(\sum_{j=1}^{n} Z_{ij} p_i \right)^{1-q} \right)^{\frac{1}{1-q}};$$

where Z is an $n \times n$ similarity matrix (Leinster & Cobbold, 2012). A similarity matrix can be expressed in terms of the distance matrix D in several ways. Leinster and Cobbold used the transformation $Z_{ij} = e^{-u\,D_{ij}}$, where u is a scaling factor. When u=1, $Z_{ij} = 1$ everywhere. Conversely, when u→∞, $Z_{ij} = 1$ and one can easily verify L_q becomes the Rényi heterogeneity in that case.

In[•]:=
```
sim[h_, u_:1] := Exp[-u dmtx[h]];
sim[h]//MatrixForm
```

Out[•]//MatrixForm=

$$\begin{pmatrix} 1 & \frac{1}{e} & e^{-\sqrt{\frac{1}{4}+h^2}} \\ \frac{1}{e} & 1 & e^{-\sqrt{\frac{1}{4}+h^2}} \\ e^{-\sqrt{\frac{1}{4}+h^2}} & e^{-\sqrt{\frac{1}{4}+h^2}} & 1 \end{pmatrix}$$

In[•]:=
```
LCIultra[q_][h_,θ_,u_:1]:= { (prob[θ].(sim[h, u].prob[θ])^(q-1))^(1/(1-q))              q≠1
                           { Product[((sim[h, u].prob[θ])^(-prob[θ]))[[i]],{i, 3}]   q==1
```

The following plots show a couple of the issues with the L_q index.

Out[•]=

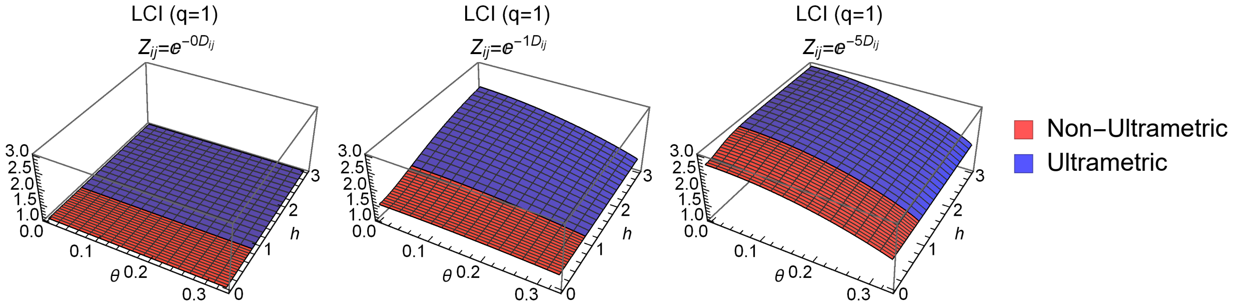

Namely, that it is particularly sensitive to the form with which the similarities are computed. As we increase the factor u, the maximal value of the L_q gradually approaches 3. However, as it does so, it appears to progressively lose sensitivity to distance. Since distances are often easier to compute than similarities, this may be a problem.

Another problem is that one only truly reaches the categorical Rényi heterogeneity when $u \to \infty$. This can be proven L_q as $h \to \infty$.

In[•]:=
```
lcilimit = Limit[FullSimplify@PowerExpand@LCIultra[1][h, θ,u], h→∞];
lcilimit = FullSimplify[lcilimit, Assumptions→{θ<1/3, u>0, e^u (1+3 θ)>-1}]
```

Out[•]=

$$\frac{3\, e^{u\left(\frac{2}{3}+\theta\right)} \left(1-3\theta\right)^{-\frac{1}{3}+\theta} \left(1+e^{u}\left(1+3\theta\right)\right)^{-\frac{1}{3}-\theta}}{\left(1+e^{u}+3\theta\right)^{1/3}}$$

Out[ ]=

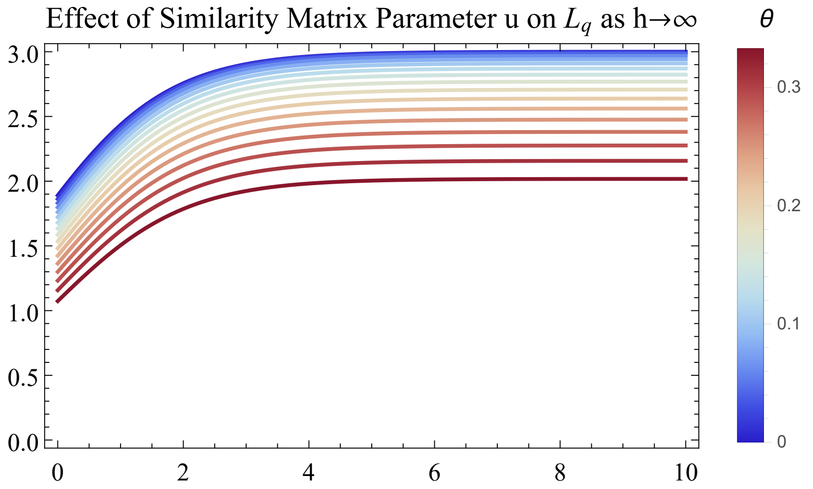

This plot shows that (as expected) the LCI will asymptotically approach a limiting value as u increases. However, the limiting value will depend on category abundance inequality θ.

Next, we show that in the limit of both $h \to \infty$ and $u \to \infty$, the upper bound on L_q decreases as a function of θ over the range [0, 1/3).

In[ ]:=

```
lcilimithu = FullSimplify[
        Limit[lcilimit, u→∞],
    Assumptions→{θ<1/3, u>0, ⅇ^u (1+3 θ)>-1}]
```

Out[ ]= $3\,(1-3\,\theta)^{-\frac{1}{3}+\theta}\,(1+3\,\theta)^{-\frac{1}{3}-\theta}$

Out[ ]=

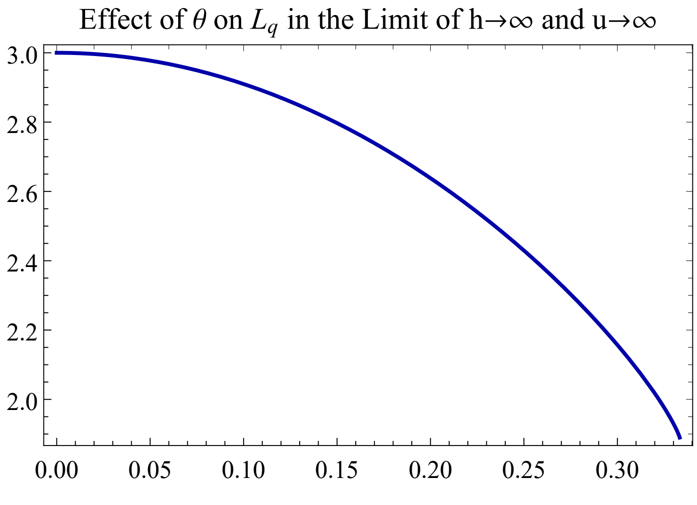

We can also show that $3\,(1-3\,\theta)^{-\frac{1}{3}+\theta}\,(1+3\,\theta)^{-\frac{1}{3}-\theta}$ is an upper bound on L_q in the limit of $h \to \infty$. We do this by showing that

$$\partial_u\left(\lim_{h\to\infty,\,u\to\infty} L_q - \lim_{h\to\infty} L_q\right) < 0$$

for finite *u*. First we compute the difference of the limit expressions.

In[ ]:=
```
dlimit = FullSimplify[
    lcilimithu-lcilimit,
    Assumptions→{θ<1/3, u>0, e^u (1+3 θ)>-1}]
```

Out[ ]= $3\left(1-3\theta\right)^{-\frac{1}{3}+\theta}\left(\left(1+3\theta\right)^{-\frac{1}{3}-\theta}-\frac{e^{u\left(\frac{2}{3}+\theta\right)}\left(1+e^{u}\left(1+3\theta\right)\right)^{-\frac{1}{3}-\theta}}{\left(1+e^{u}+3\theta\right)^{1/3}}\right)$

The result then follows from some simple algebraic manipulations.

In[ ]:=
```
dlimexpr = FullSimplify@D[ExpandAll@dlimit, u] < 0
```

Out[ ]= $-\frac{e^{u\left(\frac{2}{3}+\theta\right)}\left(1+e^{u}\right)\left(1-3\theta\right)^{-\frac{1}{3}+\theta}\left(2+9\theta\left(1+\theta\right)\right)\left(1+e^{u}\left(1+3\theta\right)\right)^{-\frac{4}{3}-\theta}}{\left(1+e^{u}+3\theta\right)^{4/3}}<0$

In[ ]:=
```
dlimexpr = FullSimplify@ApplySides[# ((1+e^u+3 θ)^(4/3)) / (e^(u (2/3+θ)) (1+e^u)) &, dlimexpr]
```

Out[ ]= $\left(1-3\theta\right)^{\frac{1}{3}+\theta}\left(2+9\theta\left(1+\theta\right)\right)\left(1+e^{u}\left(1+3\theta\right)\right)^{\frac{2}{3}+\theta}>0$

In[ ]:=
```
dlimexpr = FullSimplify@ApplySides[#/((1-3 θ)^(1/3+θ) (1+e^u (1+3 θ))^(2/3+θ)) &, dlimexpr]
```

Out[ ]= $2+9\theta\left(1+\theta\right)>0$

In[ ]:=
```
dlimexpr = ApplySides[(# - 2)/9 &, dlimexpr]
```

Out[ ]= $\theta\left(1+\theta\right)>-\frac{2}{9}$

In[ ]:=
```
FullSimplify[dlimexpr, Assumptions→{0≤θ<1/3}]
```

Out[ ]= True

□

The implications of this result are non-trivial. They ostensibly mean that the Leinster-Cobbold index will only reach true comparability with the Rényi heterogeneity when (dis)similarity is completely ignored (i.e. when the *u* parameter of the similarity function reaches ∞).

C. Numbers Equivalent Meta-Analytic Heterogeneity

In meta-analysis (DerSimonian & Laird, 1986), one is given a vector of observed effect sizes, $\mathbf{y} = (y_i)_{i=1}^n$, and variances $\mathbf{v} = (\sigma_i^2)_{i=1}^n$ for n studies. The effects are assumed to be generated under a hierarchical model depicted in the figure below.

In[ ]:=

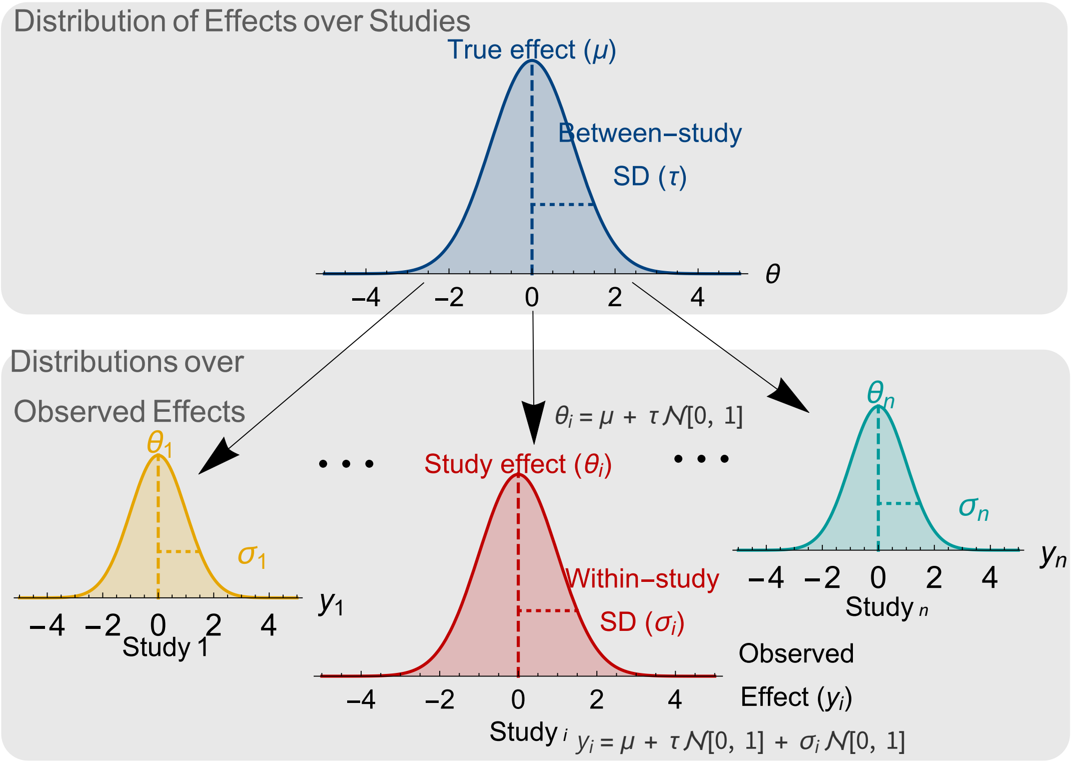

The ith study's observed effect size, y_i, is assumed to be normally distributed with mean θ_i and variance σ_i^2. The study means $\boldsymbol{\theta} = (\theta_i)_{i=1}^n$ are in turn assumed to be normally distributed with "true" mean μ (the "summary effect") and variance τ^2, which is the dispersion in study effects.

Estimation of μ, τ^2, and θ, their statistical significance, and heterogeneity proceeds as follows. This is largely based on the

Statistic	Formula	Index
Study weight	$w_i = 1/\sigma_i^2$	(1)
Weighted mean effect	$\bar{y} = \frac{\sum_{i=1}^{n} w_i y_i}{\sum_{i=1}^{n} w_i}$	(2)
Cochran's Q	$Q = \sum_{i=1}^{n} w_i (y_i - \bar{y})^2$	(3)
Degrees of freedom	$v = n - 1$	(4)
Scaling factor	$\gamma = \sum_{i=1}^{n} w_i - \left(\frac{\sum_{j=1}^{n} w_j^2}{\sum_{k=1}^{n} w_k}\right)$	(5)
Study heterogeneity	$\tau^2 = \begin{cases} \frac{1}{\gamma}(Q - v) & Q > v \\ 0 & Q \leq v \end{cases}$	(6)
Combined weights	$w_i^* = \frac{1}{\sigma_i^2 + \tau^2}$	(7)
Combined variance	$v_i^* = 1 / w_i^*$	(8)
Pooled effect	$\bar{y}^* = \frac{\sum_{i=1}^{n} w_i^* y_i}{\sum_{j=1}^{n} w_j^*}$	(9)
Standardized effect	$Z^* = \frac{\bar{y}^*}{\sqrt{\sigma_i^2 + \tau^2}}$	(10)
One tailed p – value	$p_1 = 1 - \Phi[Z^*]$	(11)
Two tailed p – value	$p_2 = 2\,(1 - \Phi[\,\lvert Z^* \rvert\,])$	(12)
Between study entropy	$H[\tau] = \frac{1}{2} \mathrm{Log}[2\,\pi\,e\,\tau^2]$	(13)
Rényi heterogeneity (q = 1)	$\Pi_1[\tau] = e^{-\frac{1}{2}\mathrm{Log}[2\,\pi\,e\,\tau^2]}$	(14)

Function implementations

In[∘]:=

```
Tmean[w_, T_]:= (∑_{i=1}^{Length[w]} w[[i]]×T[[i]]) / (∑_{j=1}^{Length[w]} w[[j]])

CochranQ[w_, T_]:= ∑_{i=1}^{Length[w]} w[[i]] (T[[i]]-Tmean[w, T])^2

DoF[T_] := Length[T]-1
ExcessVariance[w_, T_]:= CochranQ[w,T]-DoF[T]

ScalingFactor[w_]:= (∑_{i=1}^{Length[w]} w[[i]]) - ((∑_{j=1}^{Length[w]} w[[j]]^2) / (∑_{k=1}^{Length[w]} w[[k]]))

TauSquared[w_, T_]:= { (CochranQ[w, T]-DoF[T]) / ScalingFactor[w]   CochranQ[w, T]>DoF[T]
                       0                                           CochranQ[w, T]≤DoF[T]

CombinedWeights[v_, τ2_]:= 1 / (v + τ2)

CombinedVariance[w_]:= 1 / (∑_{i=1}^{Length[w]} w[[i]])

CombinedStandardError[v_]:=√v
ConfidenceInterval95[Tmean_, SE_]:= {Tmean - 1.96 SE, Tmean + 1.96 SE}
ZScore[Tmean_, SE_]:= Tmean / SE

pvalue[Z_, tails_:2]:= { SurvivalFunction[NormalDistribution[], Z]            tails==1
                         2 SurvivalFunction[NormalDistribution[], Abs[Z]]     tails==2
```

(Pseudo)Forest plot implementation

In[∘]:=

```
ForestPlot:=Function[{T, V,nullpoint, studynames, title, xlabel},
  Module[{W, data, sizelist},
    W = 1/V;
    data = Table[{T[[i]], i}, {i, 1, Length@T}];
    sizelist = Table[0.2 W[[i]]/Total[W], {i, 1, Length@T}];
    errplot =ListPlot[Table[{Around[T[[i]], Sqrt@V[[i]]], i}, {i,1 , Length@T}], PlotStyle→
    dotplot=Graphics[Table[{PointSize[sizelist[[i]]], Black,Point[data[[i]]]}, {i,1 , Lengt
    nullplot = ListLinePlot[{{nullpoint, nullpoint}, {0, Length@W+1}}, PlotStyle→Directive[R
    Show[errplot,nullplot, dotplot,
    PlotLabel→Style[title, Black, 16],
    Frame→True, FrameStyle→Directive[Black,16],
    FrameLabel→{xlabel, None},
    FrameTicks→ {Automatic, Table[{i, studynames[[i]]}, {i, Length@T}], None, None}]
]
```

C.1. Test Example

Consider the following set of studies:

In[ ]:=
```
effects = {0.3, 0.1, 0.4, 0.63, 0.22, 0.19, 0.7, -0.1};
variances = {0.01, 0.03, 0.05, 0.05, 0.05, 0.02, 0.01, 0.06};
weights = 1/variances;
```

Out[ ]=

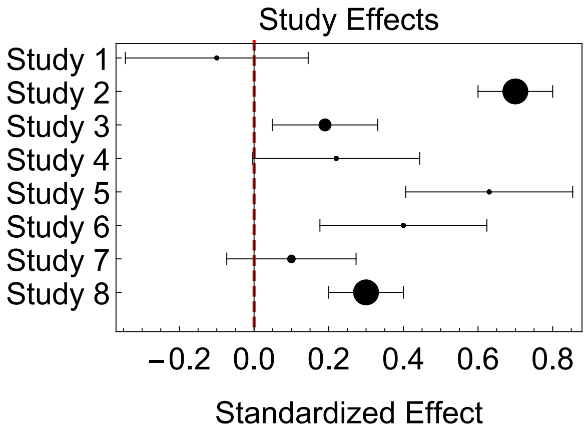

	Effect Size	Variance	Weight
**Study 8**	0.3	0.01	100.
**Study 7**	0.1	0.03	33.33
**Study 6**	0.4	0.05	20.
**Study 5**	0.63	0.05	20.
**Study 4**	0.22	0.05	20.
**Study 3**	0.19	0.02	50.
**Study 2**	0.7	0.01	100.
**Study 1**	–0.1	0.06	16.67

The traditional estimates are shown here:

In[ ]:=
```
tau = Sqrt[TauSquared[weights, effects]]
```

Out[ ]= 0.218872

In[ ]:=
```
combinedweights = CombinedWeights[variances, tau^2];
summaryeffect = Tmean[combinedweights, effects]
```

Out[ ]= 0.325376

In[ ]:=
```
combinedvar = CombinedVariance[combinedweights];
combinedSE = CombinedStandardError[combinedvar]
```

Out[ ]= 0.0990092

In[ ]:=
```
ci95 = ConfidenceInterval95[summaryeffect, combinedSE]
```

Out[ ]= {0.131318, 0.519434}

In[ ]:=
```
zscore = ZScore[summaryeffect, combinedSE]
"One tailed p-value="<>ToString[N@pvalue[zscore, 1]]
"Two tailed p-value="<>ToString[N@pvalue[zscore, 2]]
```

Out[ ]= 3.28632

Out[ ]= One tailed p-value=0.000507525

Out[ ]= Two tailed p-value=0.00101505

The following figure demonstrates the accuracy of this method based on 50 simulation runs of an 8-

study meta-analysis for increasing values of τ in the range 0.01 to 10. One can appreciate that the estimation generally falls along the line of perfect estimation in that figure.

Out[ ]=

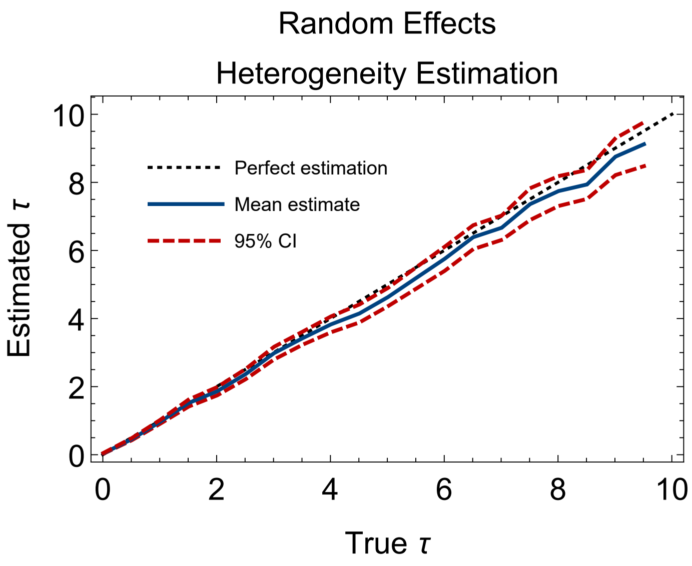

C.2. The Effective Number of Study Effects

Generally, the above estimates are used to adjust the study weights and average effect to compute the statistical significance of the summary effect. However, we will diverge from the usual treatment of random-effects meta-analysis to now demonstrate how its heterogeneity estimate can be converted to numbers equivalent. Simply put, one notes that τ^2 is the variance of a normal distribution, whose Shannon entropy is defined as $H[\tau] = \frac{1}{2}\text{Log}[2\,\pi\,e\,\tau^2]$, and whose corresponding Rényi heterogeneity is $\Pi_1[\tau] = e^{-\frac{1}{2}\text{Log}[2\,\pi\,e\,\tau^2]}$. For the same system simulated above, we show the corresponding Rényi heterogeneity below. This would correspond to the “effective number of study effects”

Out[ ]=

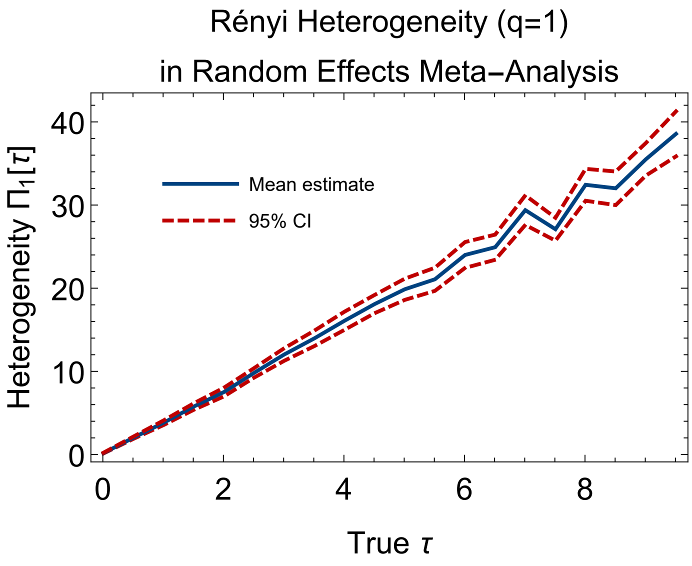

One possible criticism of this approach is the fact that the Shannon differential entropy for a continuous distribution is not a mere continuous extension of the discrete Shannon entropy. Indeed, the differential entropy can at times be negative, which should not occur for a true entropy. To this end, Jaynes (1963) showed that the appropriate extension is known as the limiting density of discrete points. Interestingly, this does not pose much of a problem for numbers equivalent heterogeneity measurements since both continuous and discrete forms are measuring the size of a distribution's base of support (Cover & Thomas, 2006).

C.2.1 Variance Violates the Axiom of Replication

One may also question why numbers equivalent would be more useful than simply reporting the variance-based meta-analytic heterogeneity index. To this end, we appeal once again to the replication principle. The replication principle effectively states that if we combine n equally heterogeneous systems with non-overlapping bases of support (i.e. event spaces), then the heterogeneity of the pooled system should be n-fold larger than the heterogeneity of any single constituent. If we use variance as the heterogeneity measure, the replication principle will be violated. Let us use a simple example with uniform distributions. Consider having n uniform distributions on the intervals,

$$\gamma = \{[\gamma_{i-1}, \gamma_i)\}_{i=1}^{n} \cap \{[\gamma_{n-1}, \gamma_n]\} \tag{5}$$

In our implementation of this here, we will simply assume $n = 4$

In[ ]:=
```
intervals[n_] := Flatten[{Table[{γi-1, γi}, {i,1, n-1}], {{γn-1, γn}}}, 1]
```

where $(\gamma_i - \gamma_{i-1}) = (\gamma_{j-1} - \gamma_j)\ \forall\ (i, j) \in \{1, 2, \ldots, n\}$ and $\gamma_{i-1} < \gamma_i\ \forall_i$. The PDF for the i'th uniform distribution is defined on the half open interval $[\gamma_{i-1}, \gamma_i)$ as follows:

In[ ]:=
```
fhalfopen[{a_, b_}][x_] := { 1/(b-a)   a≤x<b
                           { 0         True
HalfOpenUniform[{a_, b_}]:=ProbabilityDistribution[
    fhalfopen[{a, b}][x],
    {x, -∞, ∞}]
```

Pooling uniform distributions defined on the intervals in Equation 5 result in the following mixture distribution:

In[ ]:=
```
pweight[γ_] := Table[1/(Length[γ]-1), Length[γ]]
unifdist[γ_, i_] := { HalfOpenUniform[{γ[[i,1]], γ[[i,2]]}]        i<Length[γ]
                    { UniformDistribution[{γ[[i,1]], γ[[i,2]]}]    i==Length[γ]

umixdist[γ_] := MixtureDistribution[
        pweight[γ],
    Table[unifdist[γ, i], {i, 1, Length[γ]}]]
```

In[ ]:=
```
mixpdf = FullSimplify[PDF[umixdist[intervals[4]], x],
    Assumptions→{γ0<γ1<γ2<γ3<γ4, Not[x<γ1&& x≥γ2]}
]
```

Out[ ]=

$$\begin{cases} \frac{1}{4(-\gamma_0+\gamma_1)} & x \geq \gamma_0 \,\&\&\, x < \gamma_1 \\ \frac{1}{4(-\gamma_1+\gamma_2)} & x \geq \gamma_1 \,\&\&\, x < \gamma_2 \\ \frac{1}{4(-\gamma_2+\gamma_3)} & x \geq \gamma_2 \,\&\&\, x < \gamma_3 \\ \frac{1}{4(-\gamma_3+\gamma_4)} & x \geq \gamma_3 \,\&\&\, x \leq \gamma_4 \\ 0 & \text{True} \end{cases}$$

The pooled pmf will be $1/(\gamma_K - \gamma_0)$ since $(\gamma_k - \gamma_{k-1}) = (\gamma_K - \gamma_0)/4$:

In[ ]:=
```
mixpdf /. Table[(γk-γk-1)→(γ4-γ0)/4, {k, 1, 4}]
```

Out[ ]=

$$\begin{cases} \frac{1}{-\gamma_0+\gamma_4} & (x \geq \gamma_0 \,\&\&\, x < \gamma_1) \,||\, (x \geq \gamma_1 \,\&\&\, x < \gamma_2) \,||\, (x \geq \gamma_2 \,\&\&\, x < \gamma_3) \,||\, (x \geq \gamma_3 \,\&\&\, x \leq \gamma_4) \\ 0 & \text{True} \end{cases}$$

The following plot depicts an example of these distributions.

In[ ]:=

```
(* This first function constructs an array of these distributions*)
b = Range[0, 1, 0.25];
phou= Table[HalfOpenUniform[{b[[i]], b[[i+1]]}], {i, 1, Length[b]-1}];
unifdistfig = Grid[ArrayReshape[{
    Table[Plot[PDF[phou[[i]], x], {x,-0.1, 1.5},
     PlotLabel→Style[
       "𝒰["<>ToString[b[[i]]]<>","<>ToString[b[[i+1]]]<>"]",
       Black],
    Frame→True, FrameStyle→Directive[Black],
    FrameLabel→ {"x", "p[x]"},
    PlotRange→{{-0.1, 1.1}, Automatic},
  PlotStyle→ ColorData[81][i], Filling→Axis,
 ImageSize→125],
 {i, 1, Length@phou}]},
{2, 2}]];

pooleddistfig = Show[Table[
    Plot[1/4 PDF[phou[[i]], x],
    {x,-0.1, 1.5}, PlotRange→{{-0.1, 1.1}, Automatic},
    PlotStyle→ ColorData[81][i], Filling→Axis],
    {i, 1, Length@phou}],
 Frame→True, FrameStyle→Directive[Black],
FrameLabel→ {"x", "p[x]"},
PlotLabel→Style["Pooled Distributions", Black],
ImageSize→300];


unifdistgrid = Grid[{{pooleddistfig, unifdistfig}}]
```

Out[ ]=

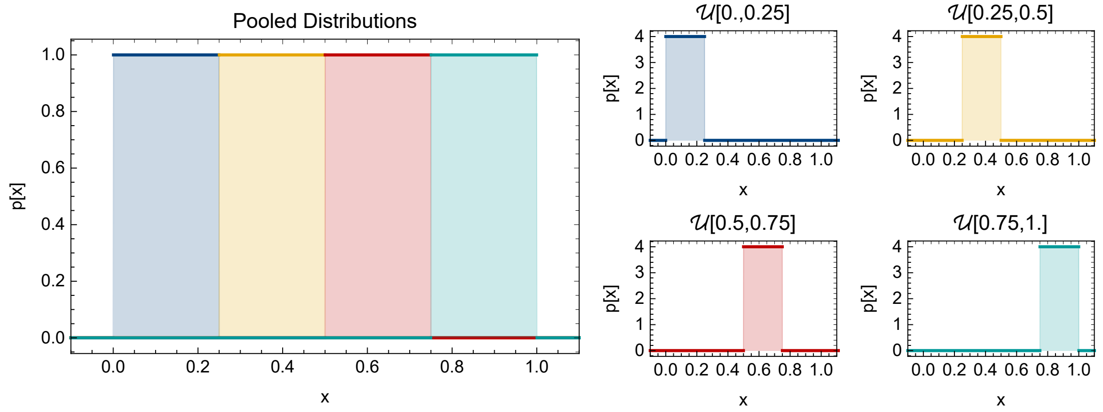

The variance for the uniform distribution on the half-open interval is the same as that of the uniform distribution on the closed interval:

In[ ]:=
```
halfovar = FullSimplify[
    Variance[HalfOpenUniform[{γi-1, γi}]],
Assumptions→{γi-γi-1>0}]
```

Out[ ]= $\frac{1}{12}(\gamma_{-1+i}-\gamma_i)^2$

In[ ]:=
```
closedvar = FullSimplify[
    Variance[UniformDistribution[{γi-1, γi}]],
Assumptions→{γi-γi-1>0}] /. γ-1+i-γi → γi-γ-1+i
```

Out[ ]= $\frac{1}{12}(-\gamma_{-1+i}+\gamma_i)^2$

Thus, the pooled variance will be as follows:

In[ ]:=
```
pooledvar = Variance[UniformDistribution[{γ0, γn}]]
```

Out[ ]= $\frac{1}{12}(-\gamma_0+\gamma_n)^2$

If we assume that the variance is a heterogeneity measure, it would satisfy the following equality under the axiom of replication:

In[ ]:=
```
rpunifvar = pooledvar == n closedvar
```

Out[ ]= $\frac{1}{12}(-\gamma_0+\gamma_n)^2 == \frac{1}{12} n (-\gamma_{-1+i}+\gamma_i)^2$

but simply rearranging shows us that it does not. Since $|\gamma_n-\gamma_0| = K|\gamma_i-\gamma_{i-1}|$, we have the following:

In[ ]:=
```
rpunifvar = rpunifvar /. (γn-γ0)→ n (γi-γi-1)
```

Out[ ]= $\frac{1}{12} n^2 (-\gamma_{-1+i}+\gamma_i)^2 == \frac{1}{12} n (-\gamma_{-1+i}+\gamma_i)^2$

In[ ]:=
```
rpunifvar = ApplySides[# 12/(-γ-1+i+γi)^2 &, rpunifvar]
```

Out[ ]= $n^2 == n$

In[ ]:=
```
TrueQ[rpunifvar]
```

Out[ ]= False

However, we can show that the continuous version of the Rényi heterogeneity,

$$\Pi_q[p] = \left(\int p[x]^q \, dx\right), \qquad (6)$$

does satisfy the axiom of replication. The Rényi heterogeneity of the uniform distribution is

In[∘]:=
```
unifhet = (∫_{γ_{i-1}}^{γ_i} (γ_i-γ_{i-1})^{-q} dx)^(1/(1-q));
unifhet = FullSimplify@PowerExpand@unifhet
```

Out[∘]= $-\gamma_{-1+i}+\gamma_i$

which conveniently describes the exact size of the base of support for the uniform model. We can now easily see that the replication principle will be satisfied.

In[∘]:=
```
repunifrenyi = (∫_{γ_0}^{γ_n} (γ_n-γ_0)^{-q} dx)^(1/(1-q)) == n (∫_{γ_{i-1}}^{γ_i} (γ_i-γ_{i-1})^{-q} dx)^(1/(1-q))
```

Out[∘]= $\left((-\gamma_0+\gamma_n)^{1-q}\right)^{\frac{1}{1-q}} == n\left((-\gamma_{-1+i}+\gamma_i)^{1-q}\right)^{\frac{1}{1-q}}$

In[∘]:=
```
repunifrenyi = PowerExpand@repunifrenyi
```

Out[∘]= $-\gamma_0+\gamma_n == n\,(-\gamma_{-1+i}+\gamma_i)$

In[∘]:=
```
repunifrenyi = repunifrenyi /. (γ_n-γ_0)→ n (γ_i-γ_{i-1})
```

Out[∘]= True

C.2.2. Numbers Equivalent of Between-Study Effects

We can now compute the Rényi heterogeneity of Gaussian distributed between-study effects. This requires us to compute the following integral:

$$\Pi_q[\tau] = \left((2\pi)^{-q/2}\,\tau^{-q}\int e^{-\frac{q(\theta-\mu)^2}{2\tau^2}}\,d\theta\right)^{\frac{1}{1-q}}, \tag{7}$$

which is that of the Gaussian pdf raised to the power q.

In[∘]:=
```
indefint = PowerExpand@Integrate[PDF[NormalDistribution[μ, τ], θ]^q, θ];
lim[x_, l_] := Limit[x, l, Assumptions→{q>0, τ>0}]
defint = (lim[indefint, θ→∞] - lim[indefint, θ→-∞])^(1/(1-q));
defint = FullSimplify@PowerExpand@defint
```

Out[∘]= $\sqrt{2\pi}\,q^{\frac{1}{2(-1+q)}}\,\tau$

We also want to compute the limit as $q \to 1$,

In[∘]:=
```
defint1 = Limit[defint ,q→1]
```

Out[∘]= $\sqrt{2e\pi}\,\tau$

and as $q \to \infty$.

In[∘]:=
```
defintinf = Limit[defint, q→∞]
```

Out[∘]= $\sqrt{2\pi}\,\tau$

The Rényi heterogeneity is thus

In[ ]:=
```
renyinorm[q_][τ_]:= { √(2 π) q^(1/(2 (-1+q))) τ   q≠1
                      √(2 ⅇ π) τ                 q==1
                      √(2 π) τ                   q==∞
```

If one estimates τ^2 using the DerSimonian & Laird method, one can then compute the "effective total number of study effects" using the above form for Rényi heterogeneity. At q=1, this would be the "effective number of typical study effects" whereas at q=2, one would have the effective "total number of common study effects," and so forth. The value of τ is computed as follows:

Showing that continuous uniform distributions satisfy the replication principle was relatively simple, since we can set those distributions up to have disjoint domains. However, all univariate Gaussians defined on the real line will have overlapping domains, so the proof becomes more difficult. Here, we simply show that the replication principle can be satisfied for two univariate Gaussians.

Recall that the Rényi heterogeneity has the following decomposition:

In[ ]:=
```
Clear[Π]
decompeq = Π_{q,γ} == Π_{q,α} Π_{q,β}
```

Out[ ]= $\Pi_{q,\gamma} == \Pi_{q,\alpha}\,\Pi_{q,\beta}$

For 2 univariate Gaussians with equal heterogeneity (and thus equal variance σ^2), the between study effect standard deviation τ is

In[ ]:=
```
τ = Sqrt[TauSquared[Table[{1/σ^2}, 2], Table[y_i, {i, 2}]]];
τ = FullSimplify@Extract[τ, {1,1,1,1,1}]
```

Out[ ]= $$\frac{1}{2}\left(-2\,\sigma^2 + (y_1 - y_2)^2\right)$$

pooled heterogeneity (where for simplicity we here assume that q=1) is

In[ ]:=
```
Π_{q,γ} = renyinorm[q][τ]
```

Out[ ]=
$$\begin{cases} \sqrt{\frac{\pi}{2}}\, q^{\frac{1}{2(-1+q)}} \left(-2\,\sigma^2 + (y_1 - y_2)^2\right) & q \neq 1 \\ \sqrt{\frac{e\pi}{2}} \left(-2\,\sigma^2 + (y_1 - y_2)^2\right) & q == 1 \\ \sqrt{\frac{\pi}{2}} \left(-2\,\sigma^2 + (y_1 - y_2)^2\right) & q == \infty \\ 0 & \text{True} \end{cases}$$

The within-study (α) heterogeneity is as follows:

In[ ]:=
$$\Pi_{q,\alpha} = \left(\frac{\sqrt{2\,\pi}\;q^{\frac{1}{2\,(-1+q)}}\left(\sum_{i=1}^{M} w_i^q\;\sigma_i\right)}{\sum_{j=1}^{M} w_j^q}\right)^{\frac{1}{1-q}}$$

Out[ ]=
$$(2\,\pi)^{\frac{1}{2\,(1-q)}}\left(\frac{q^{\frac{1}{2\,(-1+q)}}\sum_{i=1}^{M} w_i^q\,\sigma_i}{\sum_{j=1}^{M} w_j^q}\right)^{\frac{1}{1-q}}$$

We take the limit as $q \to 1$ to obtain the value for $\Pi_{1,\alpha}$. First, we move into log space and rearrange terms.

In[ ]:=
$$\text{ralphalim1 = ExpandAll@PowerExpand@Log}\left[\left(\frac{\sqrt{2\,\pi}\;q^{\frac{1}{2\,(-1+q)}}\left(\sum_{i=1}^{M} w_i^q\;\sigma_i\right)}{\sum_{j=1}^{M} w_j^q}\right)^{\frac{1}{1-q}}\right]$$

Out[ ]=
$$\frac{\text{Log}[2]}{2-2\,q}+\frac{\text{Log}[\pi]}{2-2\,q}+\frac{\text{Log}[q]}{(1-q)\;(-2+2\,q)}-\frac{\text{Log}\left[\sum_{j=1}^{M} w_j^q\right]}{1-q}+\frac{\text{Log}\left[\sum_{i=1}^{M} w_i^q\,\sigma_i\right]}{1-q}$$

In[ ]:=
$$\text{ralphalim1 = ralphalim1 /. }\frac{\text{Log}[q]}{(1-q)\;(-2+2\,q)}\to\frac{1}{1-q}\text{Log}\left[q^{\frac{1}{(-2+2\,q)}}\right]$$

Out[ ]=
$$\frac{\text{Log}[2]}{2-2\,q}+\frac{\text{Log}[\pi]}{2-2\,q}+\frac{\text{Log}\left[q^{\frac{1}{-2+2\,q}}\right]}{1-q}-\frac{\text{Log}\left[\sum_{j=1}^{M} w_j^q\right]}{1-q}+\frac{\text{Log}\left[\sum_{i=1}^{M} w_i^q\,\sigma_i\right]}{1-q}$$

Now we apply L'Hopital's rule to each term.

$$\underbrace{\frac{\text{Log}[2]}{2-2\,q}}_{\text{term1}}+\underbrace{\frac{\text{Log}[\pi]}{2-2\,q}}_{\text{term2}}+\underbrace{\frac{\text{Log}\left[q^{\frac{1}{-2+2\,q}}\right]}{1-q}}_{\text{term3}}-\underbrace{\frac{\text{Log}\left[\sum_{j=1}^{n} w_j^q\right]}{1-q}}_{\text{term4}}+\underbrace{\frac{\text{Log}\left[\sum_{i=1}^{n} w_i^q\,\sigma_i\right]}{1-q}}_{\text{term5}} \qquad (8)$$

In[∘]:=

$$\text{fracdiff}[x\_, v\_] := \frac{\partial_v \text{Numerator}[x]}{\partial_v \text{Denominator}[x]}$$

$$\text{fraclim}[x\_, v\_, l\_] := \frac{\text{Limit}[\text{Numerator}[x], v \to l]}{\text{Limit}[\text{Denominator}[x], v \to l]}$$

$$\text{lhopital}[x\_, v\_, l\_] := \text{Limit}\left[\frac{\partial_v \text{Numerator}[x]}{\partial_v \text{Denominator}[x]}, v \to l\right]$$

$$\text{term1} = \text{lhopital}\left[\frac{\text{Log}[2]}{2-2\,q}, q, 1\right];$$

$$\text{term2} = \text{lhopital}\left[\frac{\text{Log}[\pi]}{2-2\,q}, q, 1\right];$$

$$\text{term3} = \text{lhopital}\left[\frac{\text{Log}\left[q^{\frac{1}{-2+2\,q}}\right]}{1-q}, q, 1\right];$$

$$\text{term4} = \text{fracdiff}\left[\frac{\text{Log}\left[\sum_{j=1}^{M} w_j^q\right]}{1-q}, q\right] /. \left\{\frac{\sum_{j=1}^{M} \text{Log}[w_j]\ w_j^q}{\sum_{j=1}^{M} w_j^q} \to \frac{\sum_{j=1}^{M} \text{Limit}[\text{Log}[w_j]\ w_j^q, q \to 1]}{\sum_{j=1}^{M} \text{Limit}[w_j^q, q \to 1]}\right\};$$

$$\text{term5} = \text{fracdiff}\left[\frac{\text{Log}\left[\sum_{i=1}^{M} w_i^q\ \sigma_i\right]}{1-q}, q\right] /. \left\{-\frac{\sum_{i=1}^{M} \text{Log}[w_i]\ w_i^q\ \sigma_i}{\sum_{i=1}^{M} w_i^q\ \sigma_i} \to -\frac{\sum_{i=1}^{M} \text{Limit}[\text{Log}[w_i]\ w_i^q\ \sigma_i, q \to 1]}{\sum_{i=1}^{M} \text{Limit}[w_i^q\ \sigma_i, q \to 1]}\right\};$$

$$\text{ralphalim1} = \text{term1} + \text{term2} + \text{term3} + \text{term4} + \text{term5}$$

Out[∘]=

$$\frac{1}{4} - \frac{\sum_{j=1}^{M} \text{Log}[w_j]\ w_j}{\sum_{j=1}^{M} w_j} - \frac{\sum_{i=1}^{M} \text{Log}[w_i]\ w_i\ \sigma_i}{\sum_{i=1}^{M} w_i\ \sigma_i}$$

The final expression for $\Pi_{1,\alpha}$ is thus:

In[∘]:=

$$\Pi_{1,\alpha} = \text{Exp@PowerExpand}\left[\text{ralphalim1} /. \left\{w_i \to \frac{1}{\sigma^2}, w_j \to \frac{1}{\sigma^2}, \sigma_i \to \sigma\right\}\right]$$

Out[∘]= $e^{1/4}\ \sigma^4$

where the study weights are used. The between-study (β) heterogeneity is thus

In[∘]:=

$$\Pi_{1,\beta} = \frac{\text{renyinorm}[1][\tau]}{\Pi_{1,\alpha}} \text{ //FullSimplify}$$

$$\Pi_{2,\beta} = \frac{\text{renyinorm}[2][\tau]}{\Pi_{q,\alpha} /. \left\{q \to 2, w_i \to \frac{1}{\sigma^2}, w_j \to \frac{1}{\sigma^2}, \sigma_i \to \sigma\right\}}$$

Out[∘]=

$$\frac{e^{1/4} \sqrt{\frac{\pi}{2}} \left(-2\,\sigma^2 + (y_1 - y_2)^2\right)}{\sigma^4}$$

Out[∘]= $2\,\pi\,\sigma\left(-2\,\sigma^2 + (y_1 - y_2)^2\right)$

How separated do the distributions have to be in order to show that $\Pi_{q,\beta} = 2$? We solve for $\delta = |\,y_1 - y_2\,|$ at $\Pi_{q,\beta} = 2$ to yield the following relationships. First we express the β-heterogeneity in

terms of δ:

In[ ]:=

$$\text{renyibeta[q\_][}\sigma\text{\_, }\delta\text{\_] :=} \begin{cases} \frac{e^{1/4}\sqrt{\frac{\pi}{2}}\left(-2\sigma^2+\delta^2\right)}{\sigma^4} & q==1 \\ 2^{-\frac{-2+q}{2(-1+q)}}\,\pi^{\frac{q}{2(-1+q)}}\,q^{\frac{q}{2(-1+q)^2}}\,\sigma^{-1+\frac{q}{-1+q}}\left(-2\sigma^2+\delta^2\right) & q\neq 1 \end{cases}$$

In[ ]:=

$$\text{res = PowerExpand@Table}\left[\text{Extract}\left[\text{FullSimplify}\left[\text{Solve}\left[2 == \text{renyibeta[q][}\sigma, \delta], \delta\right]\right], \{2, 1, 2\}\right], \left\{q, \left\{\frac{1}{1000}, \frac{1}{2}, 1, 2, 3\right\}\right\}\right]$$

Out[ ]=

$$\left\{\sqrt{2}\sqrt{2\times 2^{1333/665334}\times 5^{500/332667}\,\pi^{1/1998}\,\sigma^{1000/999}+\sigma^2},\ \sqrt{2+8\sqrt{2\pi}}\,\sigma,\ \sqrt{2}\sqrt{\sigma^2+\frac{\sqrt{\frac{2}{\pi}}\,\sigma^4}{e^{1/4}}},\ \frac{\sqrt{1+2\pi\sigma^3}}{\sqrt{\pi}\sqrt{\sigma}},\ \sqrt{\frac{2\times 2^{1/4}}{3^{3/8}\,\pi^{3/4}\sqrt{\sigma}}+2\sigma^2}\right\}$$

The distance between y_1 and y_2 required to have a β-Rényi heterogeneity of 2 across different values of σ is shown in the following plot:

Out[ ]=

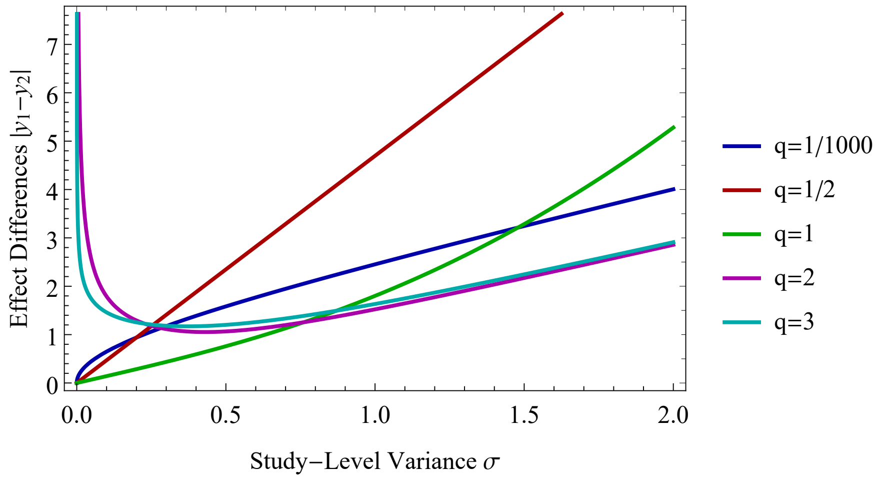

The above plot makes some interesting points. First, we note that at $q = 1/2$, the distance required between distributions to satisfy the replication principle is linear in the standard deviation ($\sqrt{2+8\sqrt{2\pi}}\ \sigma$). Secondly, as the $q > 1$, we observe convexity such that the intercept is no longer 0. At face value, this does not seem appropriate: intuition suggests that as the variances between studies becomes smaller, the difference in their means required to become “effectively categorical” should gradually approach 0.

The particularly interesting aspect of Π_q^β as a measure of between-study heterogeneity is not only that it can satisfy the replication principle, but that its units are the effective number of completely distinct study effects.

At this stage, our presentation of meta-analytic heterogeneity in the units of numbers equivalent was merely to show that this expression is possible. We are not advocating for its wider adoption at this point in time, since there are further properties that should be investigated. Namely, in the continuous domain, the heterogeneity can decrease below 1, the implications of which should be further examined. Moreover, the meaning of this heterogeneity statistic at different values of q (especially above and below 0) could warrant further investigation.

References

Chiu CH, Chao A. Distance-based functional diversity measures and their decomposition: A framework based on hill numbers. PLoS ONE. 2014;9(7).

Chao A, Chiu C-H, Jost L. Unifying Species Diversity, Phylogenetic Diversity, Functional Diversity, and Related Similarity and Differentiation Measures Through Hill Numbers. Annu Rev Ecol Evol Syst. 2014;45(1):297–324.

Cover TM, Thomas JA. Elements of information theory. 2nd ed. Hoboken, N.J: Wiley-Interscience; 2006. 748 p.

DerSimonian R, Laird N. Meta-analysis in clinical trials. Control Clin Trials. 1986;7(3):177–188.

Jaynes ET. Information Theory and Statistical Mechanics. In: Statistical Physics [Internet]. New York, NY: W.A. Benjamin, Inc.; 1963. p. 182–218. Available from: https://bayes.wustl.edu/etj/articles/brandeis.pdf

Jost L. Partitioning Diversity into Independent Alpha and Beta Components. Ecology. 2007;88(10):2427–2439.

Leinster T, Cobbold CA. Measuring diversity: The importance of species similarity. Ecology. 2012;93(3):477–489.

Rao CR. Diversity and dissimilarity coefficients: A unified approach. Theor Popul Biol. 1982;21(1):24–43.

Ricotta C, Szeidl L. Diversity partitioning of Rao’s quadratic entropy. Theor Popul Biol. 2009;76(4):299–302.

Tsallis C. Possible generalization of Boltzmann-Gibbs statistics. J Stat Phys. 1988;52(1–2):479–487.

Walker B, Kinzig A, Langridge J. Plant Attribute Diversity, Resilience, and Ecosystem Function: The Nature and Significance of Dominant and Minor Species. Ecosystems. 1999 Mar 1;2(2):95–113.

Printed by Wolfram Mathematica Student Edition
